# Eco-friendly synthesis of ZnO nanostructures from yeast strains isolated from kombucha and beetroot kwass for antimicrobial thin film applications

**DOI:** 10.1007/s00449-026-03372-0

**Published:** 2026-06-25

**Authors:** Gülden Kılıç, Gökhan Gurur Gökmen, Yogendra Kumar Mishra

**Affiliations:** 1 Department of Gastronomy and Culinary Arts, Faculty of Arts and Design, Alanya University, Antalya, Türkiye; 2 Food Quality Control and Analysis Program, Department of Food Processing, Vocational School, Alanya University, Antalya, Türkiye; 3 Smart Materials, NanoSYD, Mads Clausen Institute, University of Southern Denmark, Sønderborg, 6400 Denmark

**Keywords:** ZnO nanoparticles, Green synthesis, Antimicrobial, Amorphous, Thin films

## Abstract

The increasing demand for sustainable antimicrobial materials has accelerated the development of environmentally friendly synthesis strategies that minimize the use of hazardous chemicals and energy-intensive processes. However, the application of unconventional yeast strains for integrated nanoparticle production and thin film fabrication remains largely unexplored. In this study, yeast strains isolated from kombucha and beetroot kvass were employed for the extracellular biosynthesis of zinc oxide nanoparticles (ZnO NPs), followed by thin film fabrication, enabling systematic investigation of structure-property-antibacterial relationships. This approach provides a sustainable route for developing biologically derived antimicrobial materials with potential relevance to food and biomedical applications. ZnO NPs were synthesized and deposited as thin films via spin coating. Ultraviolet-visible spectroscopy (UV-Vis) confirmed nanoparticle formation, while scanning electron microscopy (SEM) revealed agglomerated nanoparticles and homogeneous nanoscale films. Energy-dispersive X-ray spectroscopy (EDX) verified elemental composition, and X-ray diffraction (XRD) analysis identified monoclinic and cubic crystalline phases with crystallite sizes ranging from 8.78 to 19.40 nm, whereas thin films displayed predominantly amorphous characteristics. Fourier transform infrared spectroscopy (FTIR) identified characteristic absorption bands associated with Zn–O vibrations and surface functional groups. Water contact angle (WCA) analysis indicated hydrophilic surfaces. ZnO NPs exhibited pronounced antibacterial activity against *Staphylococcus aureus* ATCC 6538P and *Escherichia coli* O157:H7. Although thin films produced smaller inhibition zones, complete bacterial inactivation was achieved within 2 h under direct-contact conditions. These findings demonstrate the potential of biosynthesized ZnO nanostructures as sustainable antimicrobial platforms.

**Graphical abstract:**

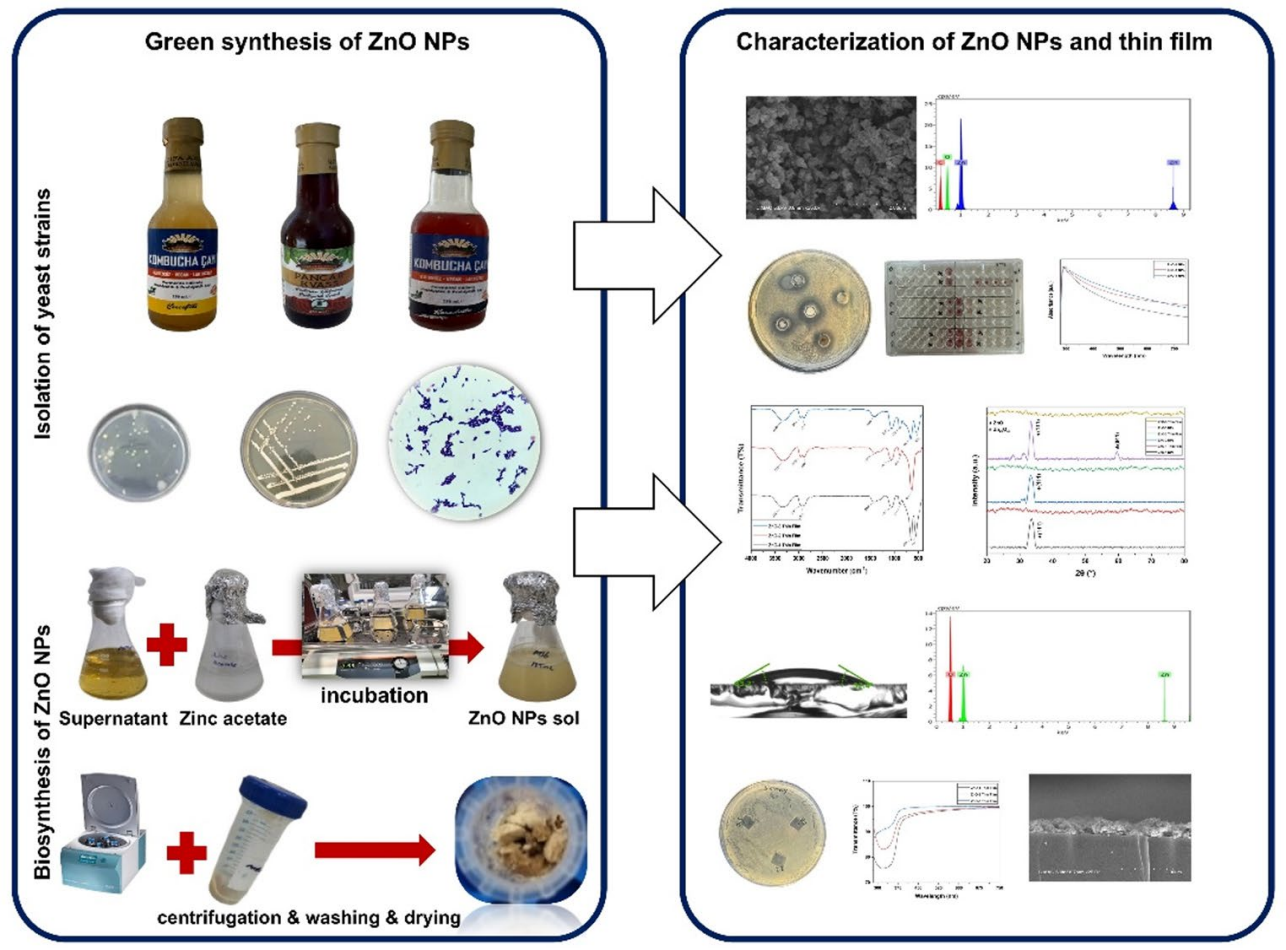

**Supplementary Information:**

The online version contains supplementary material available at 10.1007/s00449-026-03372-0.

## Introduction

Nanotechnology involves the controlled design and manipulation of materials at the nanoscale, where unique physicochemical properties emerge compared to their bulk counterparts [1]. Nanoparticles (NPs), characterized by at least one dimension in the 1–100 nm range, can be fabricated from a wide range of metallic and metal oxide materials, especially transition element-based systems, through both top-down and bottom-up approaches [2, 3]. Nanoparticle (NP) fabrication can generally be categorized into bottom-up and top-down approaches; the former is based on the controlled assembly of atoms or molecules into nanoscale structures, whereas the latter involves the size reduction of bulk materials through various physical or energy-assisted processes [4]. These approaches enable fine-tuned regulation of NP morphology and stability, thereby expanding their utility for diverse technological applications [5]. Nevertheless, such synthetic methodologies often demand costly instrumentation and hazardous reagents, raising concerns regarding human and environmental toxicity while also constraining scalability [2, 6]. Accordingly, increasing attention has been directed toward the design of synthesis methods that combine environmental sustainability with economic viability. In parallel with these principles, nanotechnology has increasingly permeated a wide range of real-world applications across diverse sectors. In biomedical applications, NPs are widely used in targeted drug delivery, diagnostic imaging, antimicrobial coatings, and controlled therapeutic systems [7–10]. In the food sector, nanostructured systems are employed in active packaging, spoilage detection through nanosensors, antimicrobial surface coatings, and nutrient enhancement [11–14]. From an environmental perspective, nanotechnology contributes to processes such as water remediation, elimination of pollutants, and the advancement of membrane-driven filtration technologies [15–17]. Beyond industrial and biomedical uses, nanomaterials are increasingly integrated into everyday consumer products, where they provide benefits such as enhanced protection against UV radiation, improved material resilience, and advanced functional performance [18–20].

Green synthesis has emerged as a sustainable alternative for nanoparticle production, offering reduced environmental impact, enhanced biocompatibility, and elimination of toxic reagents [21]. In addition to environmental benefits, green synthesis approaches enable the incorporation of biologically derived capping agents, which can enhance NP stability, reduce toxicity, and improve functional performance compared to chemically synthesized counterparts [22, 23]. Unlike conventional methods, it eliminates the need for hazardous reagents and harsh processing conditions, instead employing biological agents such as microorganisms (bacteria, fungi, algae, or viruses) and plant-derived compounds (alkaloids, flavonoids, proteins, terpenoids, etc.) to mediate NPs formation [24, 25]. Typically, metal salts undergo biological reduction, yielding NPs that are subsequently characterized for application [26]. Microorganisms facilitate this process through biochemical pathways, most notably NADH-dependent enzymatic reduction, which can occur intracellularly or extracellularly [27, 28]. While both routes are effective, extracellular synthesis is often preferred due to its simplicity and efficiency [29].

Probiotic microorganisms, widely applied in food fermentations and nutraceuticals, have emerged as promising candidates for NP biosynthesis. Yeasts, isolated from fermented beverages such as kombucha and beetroot kvass are rich in bioactive metabolites, including organic acids, enzymes, and phenolic compounds, which not only facilitate NP synthesis but may also contribute to enhanced antimicrobial activity and biocompatibility of the resulting nanomaterials [22, 23]. Species such as lactic acid bacteria (LAB), acetic acid bacteria (AAB), and yeasts, which are traditionally used as starter cultures in products like pickles, vinegar, wine, beer, kombucha, shalgam, and kefir, serve as effective biological platforms for NP generation [30, 31]. Strains including *Lactobacillus* sp., *L. acidophilus*, *Glucanoacetobacter kombuchae*, and *Saccharomyces cerevisiae* have been employed to synthesize NPs such as Ag, Fe_2_O_3_, and Se [32–35]. Biologically derived NPs have attracted increasing interest due to their versatility, with potential uses extending to medical treatments, agricultural applications, pharmaceutical delivery systems, and the food industry [36, 37].

The unique combination of antimicrobial activity, UV-blocking behavior, and elevated surface area makes ZnO NPs highly attractive for a range of functional applications [13]. The antibacterial activity of ZnO NPs is associated with multiple mechanisms, including ROS generation, membrane disruption, and Zn^2+^ ion release [38, 39]. Although a wide range of metal and metal oxide NPs such as Ag, TiO_2_, Cu, and MgO are commercially available, each system presents inherent limitations including high cost (Ag), potential cytotoxicity (Ag, Cu), limited stability (Cu), or reduced antimicrobial efficiency under certain conditions (TiO_2_ without UV activation). The combination of economic feasibility, chemical stability, broad antimicrobial performance, and recognized biocompatibility makes ZnO NPs particularly advantageous compared to many other nanomaterials [22, 40]. A comparison of commonly used metal oxide nanomaterials and their advantages and limitations is presented in Table 1. Furthermore, ZnO is classified as “generally regarded as safe” (GRAS), making it particularly suitable for food and biomedical applications where safety is a primary concern [39, 41]. As summarized in Table 1, although several metal oxide nanomaterials exhibit antimicrobial activity, ZnO NPs provide a unique balance between antibacterial efficiency, biocompatibility, and environmental sustainability. In particular, the green synthesis approach employed in this study enables the production of defect-rich, biomolecule-capped ZnO nanostructures with tunable physicochemical and functional properties [41]. ZnO NPs exhibit tunable cytocompatibility that varies with size and dosage, and when applied under controlled conditions, they can maintain low toxicity toward mammalian cells, reinforcing their applicability in biomedical fields [23, 38]. Although the hexagonal wurtzite phase represents the most stable crystalline form of ZnO, alternative metastable cubic structures, such as zinc blende and, more rarely, rock-salt configurations, may develop depending on the synthesis environment and processing conditions [42, 43]. The occurrence of these cubic phases can be attributed to a combination of synthesis-related factors, such as confined growth at the nanoscale, relatively low processing temperatures, and the involvement of biomolecule-driven nucleation processes. In green synthesis systems, particularly those based on biological or plant-derived media, organic molecules can influence crystal growth pathways and contribute to the stabilization of non-wurtzite configurations [44, 45]. Compared to the conventional wurtzite phase, cubic ZnO nanostructures exhibit distinct surface energetics, defect states, and electronic properties, which can significantly affect their optical absorption characteristics, excitonic behavior, catalytic performance, and antimicrobial activity. Therefore, the ability to stabilize such metastable cubic phases offers a promising strategy for tuning material functionality and has increasingly attracted attention in the context of green-synthesized and solution-processed nanomaterials [46–48]. In this context, the emergence of cubic ZnO phases in our study is consistent with previous reports indicating that biological reducing and capping agents may direct crystallization pathways toward alternative polymorphic states. With the continued rise of foodborne infections and antimicrobial resistance, there is a growing demand for safe antimicrobial approaches, and biogenic ZnO NPs have emerged as a viable and sustainable option [13]. Conventional antimicrobial strategies face several challenges, including the emergence of antibiotic-resistant microorganisms, limited durability of antimicrobial coatings, and potential toxicity associated with chemical disinfectants. These limitations necessitate the development of alternative antimicrobial systems that are both effective and safe for long-term use [22, 38]. Recent studies have demonstrated the green synthesis of ZnO NPs using microorganisms including *Bacillus* sp., *B. subtilis*, *Lactiplantibacillus plantarum*, *L. gasseri*, *Lactococcus lactis*, *Pediococcus* sp., *Saccharomyces cerevisiae*, and *Weissella species* which yielded NPs with notable anticancer, antimicrobial, and anti-inflammatory properties [28, 29, 49–58]. While microbial routes for ZnO and other NPs synthesis have been widely examined, the use of yeasts from traditional fermented beverages such as kombucha and beetroot kvass remains largely unexplored. Despite the extensive body of research on the green synthesis of ZnO NPs, the role of non-conventional yeasts from fermented beverages as bio-factories for ZnO nanostructures remains largely unexplored.

**Table 1:** Comparative evaluation of metal oxide-based nanomaterials reported in previous studies in terms of synthesis strategies, physicochemical properties, antimicrobial performance, and application potential

Material	Synthesis route	Key properties	Antimicrobial efficiency	Advantages	Limitations	Application	References
Ag NPs	Chemical/green	High surface reactivity	Very high (broad spectrum)	Strong antibacterial	High toxicity, expensive	Medical	[3, 22, 40]
TiO_2_	Chemical	Photocatalytic	Moderate (UV-dependent)	Stable, low cost	Requires UV activation	Coatings	[40, 141]
CuO	Chemical/green	High ion release	High	Cost-effective	Cytotoxicity risk	Industrial	[22, 141]
MgO	Chemical	Alkaline surface	Moderate	Low toxicity	Lower efficiency	Food	[2]
ZnO	Chemical/green	ROS and Zn^2+^ release	High	Biocompatible, GRAS	Defect-dependent	Food/biomedical	[13, 23, 38, 39, 41]
ZnO/TiO_2_	Composite	Enhanced photocatalysis	High	Synergistic effect	Complex synthesis	Coatings	[141]
CuO/TiO_2_	Composite	ROS + ion release	High	Improved activity	Toxicity concerns	Industrial	[141]
ZnO/CuO	Composite	Dual mechanism	Very high	Synergistic antibacterial	Stability issues	Hybrid coatings	[141]

The present study aimed to synthesize ZnO NPs using extracellular extracts obtained from yeast isolates recovered from fermented beverages and to evaluate their structural, physicochemical, and antibacterial properties together with their corresponding thin film forms. Unlike previous studies primarily focused on NPs production, this work introduces, for the first time, the extracellular biosynthesis of ZnO NPs employing *Zygosaccharomyces bailii* and *Brettanomyces anomalus* isolated from kombucha and beetroot kvass. In addition, nanoparticle synthesis was integrated with spin-coated thin film fabrication to establish direct structure-property-activity relationships. By linking microbial origin, crystallographic characteristics, wettability behavior, and antibacterial performance, this study provides new insights into the development of biologically derived antimicrobial coatings with potential applications in food safety and biomedical fields.

## Materials and methods

### Enumeration, isolation and identification of yeast strains from fermented beverages

This study utilized a range of spontaneously fermented beverages, including kombuchas (prepared with ginger and black mulberry), and beetroot kvass fermented from red beetroot as wild-type yeast strain sources. All beverage samples were obtained from a local producer in Alanya, Antalya (Türkiye). At each collection point, the beverages were preserved in their original bottles, maintained at 4 °C during transportation, and delivered to the laboratory within 24 h.

Serial dilutions of the fermented beverages were prepared and plated in duplicate on selective growth media for microbial enumeration. Mold and yeast populations were quantified using the pour plate method on Malt Extract Agar (MEA, pH 5.6 ± 0.2, Merck, Germany), acidified with 10% lactic acid (Merck, Germany). Plates were incubated at 25 °C for 3–5 days following FDA-BAM [59] guidelines. After incubation, three representative yeast colonies were randomly selected, purified by streak plating on MEA, and maintained at -18 °C in Potato Dextrose Broth (PDB, pH 5.6 ± 0.2, CondaLab, Spain) supplemented with 50% (v/v) glycerol for long-term preservation.

The internal transcribed spacer (ITS) region (ITS1-5.8 S-ITS4 rDNA) of the yeast isolates was amplified using ITS1 (5′-TCC GTA GGT GAA CCT GCG G-3′) and ITS4 (5′-TCC TCC GCT TAT TGA TAT GC-3′) primers. Genomic DNA was extracted with the High Pure PCR Template Preparation Kit (Sigma-Aldrich), and PCR reactions were conducted using the Xpert Fast Hotstart 2X Mastermix Kit (Grisp) with universal ITS primers. Amplicons were sequenced by Sanger sequencing, and resulting sequences were compared to reference strains using BLAST at the National Center for Biotechnology Information (NCBI). Identified sequences were deposited in GenBank under accession numbers PV200289 (M1), PV200294 (M2), and PV219289 (M3) [60].

**Fig. 1:**
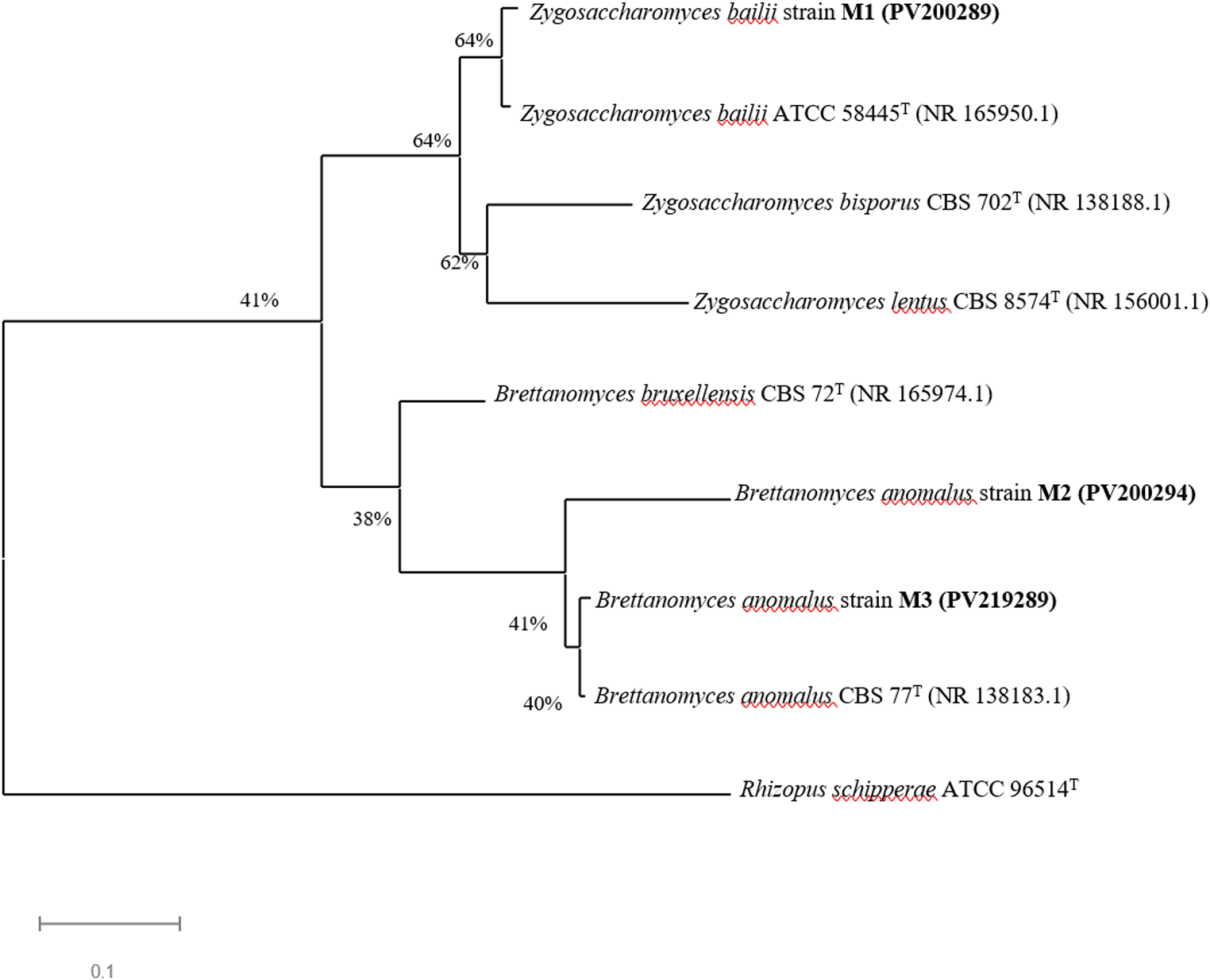
Phylogenetic relationships between yeast strains (M1, M2 and M3) isolated from fermented beverages and type strains

Phylogenetic analyses were performed using MEGA X [61]. The evolutionary history was reconstructed by the Neighbor-Joining method [62], with evolutionary distances estimated using the Tamura-Nei model [63, 64]. Optimal trees were generated for the yeast isolates (Fig. 1) using nine nucleotide sequences. As references, type strains (*Zygosaccharomyces bailii* ATCC 58445, *Zygosaccharomyces bisporus* CBS 702 and CBS 8574, *Brettanomyces bruxellensis* CBS 72, *Brettanomyces anomalus* CBS 77) and the outgroup *Rhizopus schipperae* ATCC 96,514 were retrieved from the NCBI database and included to illustrate phylogenetic relationships.

### Biosynthesis of ZnO NPs by isolated yeast strains

ZnO NPs were synthesized using beneficial microorganisms derived from fermented beverages, specifically three yeast strains (M1, M2, and M3). Yeast cultures were activated in Potato Dextrose Broth (PDB) at 25 °C for 48–72 h, followed by centrifugation for 15 min at 10,000 rpm to obtain cell-free supernatants. These supernatants served as the biological medium for extracellular NP synthesis, following the protocol of Ramesh et al. [65] with minor modifications.

For each reaction, 100 mL of culture supernatant (pH 7.5) was mixed with 50 mL of 0.1 M zinc acetate [Zn(CH_3_CO_2_)_2_] in a 250 mL erlenmeyer flask. The mixtures were incubated at 37 °C in a shaking incubator at 150 rpm for 24 h. NP formation was indicated by a distinct color shift of the solutions from light brown to pale or deep white. The reaction mixtures were subsequently centrifuged at 10,000 rpm for 15 min at 4 °C, and the supernatant was discarded. To ensure purity, the pellet was repeatedly washed with distilled water and centrifuged three additional times under identical conditions. The resulting NP biomass was oven-dried at 40 °C for 18–24 h, yielding fine ZnO NP powders, which were collected and stored for subsequent characterization [65].

### Spin coating of ZnO NPs

Spin coating was selected as a simple, reproducible, and cost-effective approach for fabricating homogeneous ZnO thin films with controlled surface coverage. In the present study, this technique was employed to transform biosynthesized ZnO NPs into surface-bound coatings and to investigate the influence of thin-film architecture on physicochemical and antibacterial properties. The spin-coating technique was employed to fabricate ZnO thin films on glass substrates using a Laurell WS-400BZ-6NPP/LITE (REV. MS) spin coater. The procedure was adapted from the method reported by Malpure et al. [66], with minor modifications to suit the present experimental conditions. Glass microscope slides were cut into 2 × 2 cm pieces and subjected to sequential ultrasonic cleaning for 15 min each using acetone (Merck, Germany), isopropanol (Merck, Germany), and distilled water. ZnO NPs were then dispersed in 70% ethanol at a concentration of 1% (w/v) and used as coating solutions. During spin coating, ZnO suspensions were dispensed onto glass substrates and spun at 3,000 rpm for 1 min under ambient conditions. The resulting films were dried on a hot plate at 90 °C for 10 min to remove residual solvent. This coating procedure was repeated three times, after which the samples were annealed in a furnace (Nabertherm, Germany) at 400 °C for 1 h, yielding ZnO thin films.

### Structural characterization of ZnO NPs and thin film

The optical characteristics of the synthesized ZnO NPs were examined using UV-Vis spectroscopy (Thermo Evolution 3000) within the 300–700 nm wavelength range at a resolution of 2 nm, with maximum absorbance peaks recorded [67].

Morphological and structural features were assessed through SEM-EDX (Hitachi S-4800). Crystallographic phase and structural information were determined by XRD analysis using a Rigaku SmartLab 3 kW system. In addition, FTIR spectroscopy (Shimadzu IRAffinity-1 S HATR 10) was employed to identify potential biomolecules involved in Zn^2+^ ion reduction, as well as to evaluate the formation and stability of the ZnO NPs [68].

The surface wettability of ZnO thin films was determined via static water contact angle measurements following the methodology described by Kılıç [69]. Briefly, measurements were performed under controlled conditions using the sessile drop technique, and the reported values represent the average of multiple measurements taken from different regions of independently prepared samples to ensure reproducibility and statistical reliability.

### Assessment of antibacterial activity of ZnO NPs

*E. coli* O157:H7 ATCC 35150 and *S. aureus* ATCC 6538P were employed as representative pathogenic strains. These bacterial cultures were procured from the Food Microbiology Laboratory of the Department of Food Engineering at Ege University (Izmir, Türkiye), and were cultivated in Tryptic Soy Broth (TSB; pH 7.3 ± 0.2, Merck, Germany) at 37 °C for 24 h. Overnight cultures grown in TSB under the same conditions were subsequently harvested and suspended in Phosphate Buffered Saline (PBS; 8 g/L NaCl, 1.42 g/L Na_2_HPO_4_, 245 mg/L KH_2_PO_4_, 200 mg/L KCl; pH 7.4 ± 0.2) to prepare the inocula. The optical density of the bacterial suspensions was adjusted to match a McFarland standard of 0.5, corresponding to approximately 8 log CFU/mL, by appropriate dilution in PBS.

Three distinct ZnO NPs suspensions (ZnO-1 NPs, ZnO-2 NPs, and ZnO-3 NPs) were formulated in distilled water at a final concentration of 1% (w/v). To ensure homogenous dispersion, each suspension was subjected to vortex mixing for 15 min, followed by ultrasonication using a Bandelin Sonorex device for an additional 30 min.

### Agar-well diffusion method

The effect of ZnO NPs was assessed with a modified version of the agar well diffusion technique originally described by Argyri et al. [70]. Briefly, 100 µL of each bacterial test suspension was aseptically spread onto the surface of Nutrient Agar (NA; pH 7.0 ± 0.2, Merck, Germany) using the spread plate method. The inoculated plates were then allowed to dry under sterile conditions within a biosafety cabinet (Thermo Safe 2020) for approximately 15 min. Subsequently, 100 µL of each ZnO NPs suspension was carefully dispensed into wells (6 mm in diameter) that had been aseptically bored into the agar medium. After 24 h of incubation at 37 °C, antibacterial effectiveness was evaluated by recording the size of the clear inhibition zones surrounding each well, which reflect suppression of bacterial growth.

### Determination of the minimum inhibitory concentration (MIC) and minimum bactericidal concentration (MBC)

The MIC of the ZnO NPs was determined using the broth microdilution method in 96-well microplates [71]. Initially, 80 µL of double-strength Mueller-Hinton Broth (pH 7.4 ± 0.2, Merck, Germany) was dispensed into each well. Thereafter, 80 µL of the ZnO NPs suspension was added to the wells in the first column, followed by a two-fold serial dilution across the plate to yield final NP concentrations of 5.00–0.0078 mg/mL (w/v), respectively. Subsequently, 20 µL of the bacterial inoculum was introduced into each well, resulting in a final volume of 100 µL per well. The microplates were then incubated at 37 °C for 24 h.

Post-incubation, bacterial metabolic activity was assessed by adding 20 µL of 0.5% aqueous 2,3,5-triphenyl tetrazolium chloride (TTC; Merck, Germany) to each well, followed by a further 30-min incubation at 37 °C. The MIC was defined as the lowest concentration of ZnO NPs that completely inhibited visible bacterial growth, as indicated by the absence of red color development. To determine the MBC values, aliquots from wells corresponding to and above the MIC were subcultured onto NA and incubated at 37 °C for 24 h. The MBC was identified as the lowest concentration at which no colony formation was observed, indicating ≥ 99.99% reduction in the original bacterial population and confirming bactericidal activity, as described by Kışla et al. [72].

### Direct contact test

The antibacterial performance of ZnO thin films (ZnO-1, ZnO-2, and ZnO-3) was investigated using a direct contact assay in accordance with the methodology reported methods by Beyth et al. [73] and Akan et al. [74]. Briefly, bacterial suspensions were applied onto the film surfaces and incubated under controlled conditions. Viable cell counts were determined at defined time intervals, and the number of surviving bacteria was quantified using standard plate count methods. All analyses were performed in replicate to ensure the reliability and reproducibility of the results.

### Modified Kirby-Bauer test

Bacterial test cultures were cultivated in Tryptone Soya Broth (TSB, pH 7.3 ± 0.2; Oxoid, UK) at 37 °C for 24 h, after which the cell suspensions were adjusted to a turbidity equivalent to a 0.5 McFarland standard using sterile PBS. Subsequently, 100 µL aliquots of the standardized bacterial suspensions were evenly spread onto NA plates and allowed to air dry aseptically within a biosafety cabinet for 15 min. Following placement in an inverted orientation onto the agar medium, the thin films were incubated for 24 h at 37 °C. For comparison, oxacillin antibiotic discs (1 µg/disc) were used as positive controls against *S. aureus* ATCC 6538P, while gentamicin discs (10 µg/disc) were used as positive controls against *E. coli O157:H7*. All experiments were performed in triplicate for each sample group to ensure reproducibility. Post incubation, the antibacterial effect was assessed by measuring the diameter of the inhibition zones (in millimeters) surrounding the films, following the protocol established by Kışla et al. [72].

### Statistical analysis

All statistical evaluations were performed with IBM SPSS software, considering *p* < 0.05 as the threshold for significance. Group differences were analyzed using one-way ANOVA followed by Duncan’s multiple range test, and independent-sample t-tests were applied when appropriate.

## Results

### Isolation and molecular identification of yeast strains

In this study, spontaneously fermented beverages (kombuchas and beetroot kvass) were utilized as sources of yeast as beneficial microorganisms. The yeast populations in these beverages ranged from 3.74 to 5.07 log CFU/mL. Morphological examination of yeast colonies grown on PDA medium was carried out by considering parameters such as colony size, form, and color characteristics. From the fermented beverages, a total of 9 yeast isolates (2 from kombucha with ginger, 5 from kombucha with black mulberry, and 2 from beetroot kwass produced from red beetroot) were obtained, reflecting the yeast counts. Among these, three isolates (M1 from kombucha prepared with ginger, M2 from kombucha prepared with black mulberry, and M3 from beetroot kwass produced from red beetroot) were randomly selected for the biosynthesis of ZnO NPs, given their established status as beneficial microorganisms in fermented matrices.

Based on ITS1-5.8 S-ITS2 rDNA sequence analysis, the M1 strain was classified as *Zygosaccharomyces bailii*, whereas M2 and M3 were identified as *Brettanomyces anomalus*. Following the submission of their nucleotide sequences to GenBank, NCBI, these strains were assigned the accession numbers PV200289 (M1), PV200294 (M2), and PV219289 (M3). Phylogenetic analysis further confirmed their taxonomic placement: M1 clustered closely with *Z. bailii* ATCC 58,445, while M2 and M3 were closely related to *B. anomalus* CBS 77, serving as the respective type strains (Fig. 1).

Within the genus *Zygosaccharomyces*, *Z. bailii* is a facultatively anaerobic yeast notable for its remarkable tolerance to weak-acid food preservatives. Although it can act as a spoilage organism in acidic, sugar-rich foods and beverages, it also has beneficial enological applications, such as enhancing wine quality by increasing polysaccharide content [75]. Members of the genus *Brettanomyces* are non-sporulating (anamorphic), facultatively anaerobic, and Crabtree-positive yeasts [76, 77]. Specifically, *B. anomalus* can hydrolyze sucrose into glucose and fructose via invertase, ferment glucose to ethanol, and subsequently oxidize ethanol to acetic acid, or convert glucose directly into acetic acid [78, 79]. The production of these metabolites may confer antibacterial properties. Both *Z. bailii* and *B. anomalus* are commonly present in a variety of foods, including fruits, vegetables, fermented beverages, and alcoholic drinks. Moreover, these yeasts have been reported to play a role in producing low-alcohol, low-sugar functional beverages with potential health benefits [80].

### Green synthesis of ZnO NPs by yeast strains

In the present study, ZnO NPs were synthesized through the reduction of aqueous zinc acetate using cell-free supernatants obtained from yeast strains identified as *Z. bailii* and *B. anomalus*. The formation of ZnO NPs was confirmed and characterized using a range of analytical techniques. The appearance of pale yellow to white precipitates indicated the biotransformation of zinc ions into ZnO NPs mediated by the microbial supernatants [81]. Previous studies have demonstrated that various yeast species, including *Candida albicans*, *Saccharomyces boulardii*, *S. cerevisiae*, and *Pichia kudriavzevii*, are capable of synthesizing ZnO NPs, often mediated by extracellular biomolecules such as proteins and polysaccharides [52, 82–84]. However, to our knowledge, the biosynthesis of ZnO NPs using *Z. bailii* and *B. anomalus* has not been previously reported. This novelty suggests that less commonly studied yeasts may serve as valuable microbial factories for NP synthesis, potentially broadening the scope of green nanotechnology applications. Moreover, the ability of these strains to facilitate extracellular synthesis highlights their practical advantages, since downstream processing is less complex compared to intracellular routes.

The biosynthesis of ZnO NPs by the isolated yeast strains can be explained through a biomolecule-mediated extracellular mechanism. In such systems, microorganisms secrete a variety of bioactive compounds, including enzymes, proteins, polysaccharides, and electron-shuttling molecules, which actively participate in nanoparticle formation. Initially, Zn^2+^ ions interact with negatively charged functional groups present in these biomolecules (such as carboxyl, amine, and amide groups), leading to electrostatic attraction and complex formation. Subsequently, enzymatic reduction occurs, primarily mediated by NADH-dependent reductase enzymes, which facilitate electron transfer from NADH to metal ions, resulting in their conversion into nanoscale ZnO structures [85]. In extracellular synthesis pathways, this reduction step is often attributed to nitrate reductase and other redox-active enzymes secreted into the surrounding medium, enabling the transformation of metal ions into stable nanoparticle nuclei [86]. Following nucleation, growth and stabilization of NPs are governed by capping effects, where proteins and other biomolecules adsorb onto the NP surface, preventing excessive aggregation and controlling particle size and morphology. FTIR findings in the present study, indicating the presence of hydroxyl, alkane, and aromatic functional groups, further support the involvement of these biomolecules in both reduction and stabilization processes. Moreover, yeast cells are known to possess detoxification mechanisms involving metal-binding ligands such as glutathione, metallothioneins, and phytochelatins, which can bind and transform toxic metal ions into less reactive NP forms [87]. This biological response not only facilitates NP formation but also contributes to their stabilization and dispersion. Therefore, the overall biosynthesis mechanism can be summarized as a sequential process involving ion binding, enzymatic reduction, nucleation, growth, and biomolecule-mediated stabilization, which is consistent with previously reported microbial nanoparticle synthesis pathways [52, 84].

### UV-Vis spectrum of ZnO NPs and thin films

**Fig. 2:**
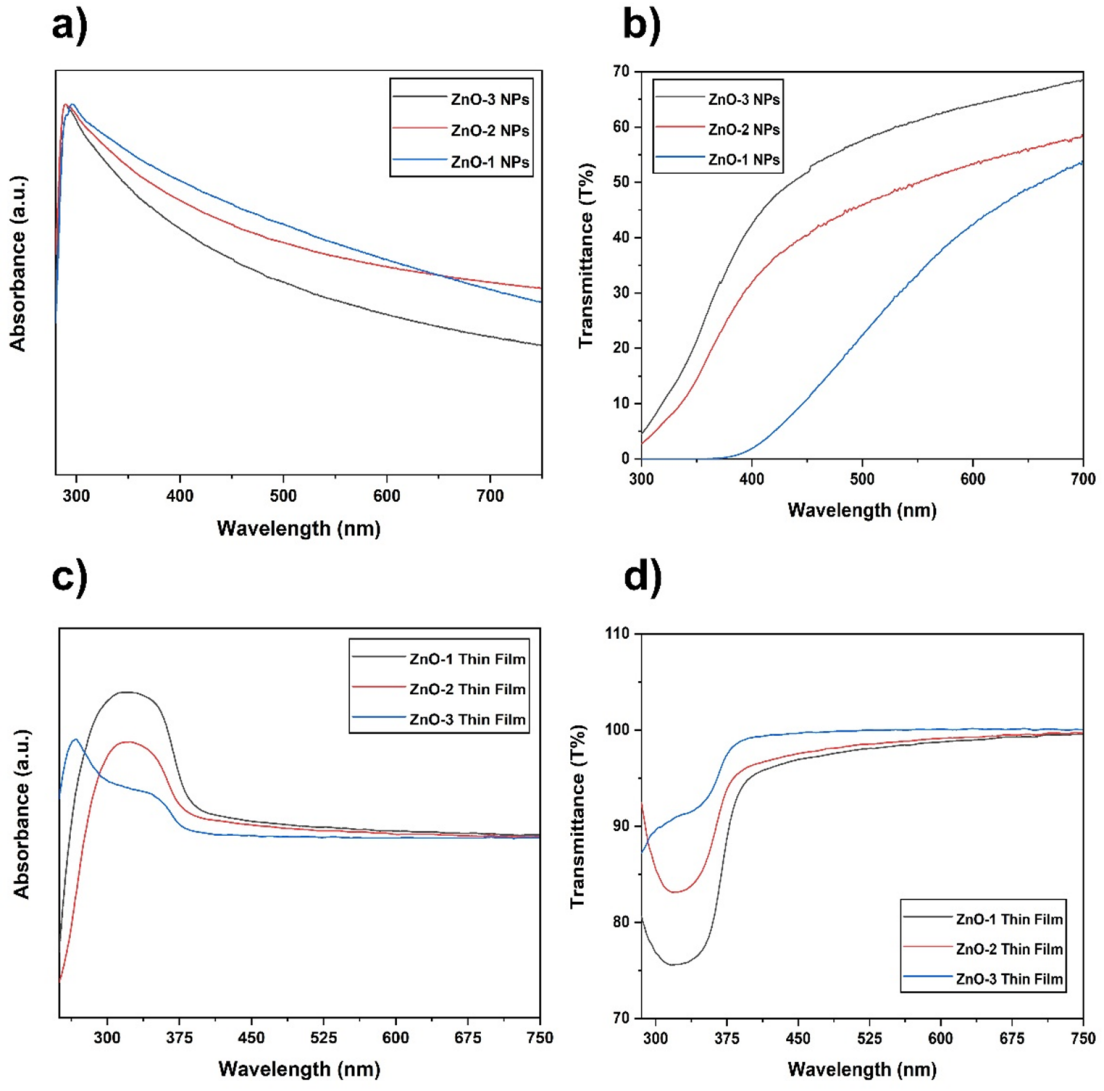
UV-Vis spectra of ZnO NPs (**a**, **b**) and thin films (**c**, **d**)

UV-Vis analysis provided evidence for the successful synthesis of ZnO NPs obtained through green routes, as well as the formation of their associated thin film structures. ZnO NPs produced by *Z. bailii* M1, *B. anomalus* M2, and *B. anomalus* M3 strains, along with the thin films fabricated from these NPs, displayed characteristic absorption peaks centered around 300 nm (Fig. 2a and c). Figure 2b and d present the transmittance spectra of ZnO NPs and corresponding thin films. Although all samples exhibited absorption features around 300 nm, subtle variations in peak position and transmittance behavior suggest differences in their optical characteristics. Slight spectral shifts observed among ZnO-1, ZnO-2, and ZnO-3 samples may indicate changes in crystallite size, defect density, and surface chemistry arising from strain-specific biomolecules involved during biosynthesis [88, 89]. Such redshift or blueshift behavior has frequently been associated with alterations in particle size distribution, quantum confinement effects, and the presence of defect-rich surface states in ZnO nanostructures [84]. In the visible region (400–700 nm), ZnO-3 thin films exhibited higher transmittance than ZnO-1 and ZnO-2 films, suggesting improved optical transparency and a lower degree of light scattering. This behavior may be associated with differences in film uniformity, nanoparticle packing density, and defect distribution. Previous studies have shown that structural defects and surface states can modify optical transitions and significantly influence absorption behavior in ZnO-based materials [89]. Therefore, the observed optical differences among the samples likely originate from variations in crystallographic structure and strain-dependent biosynthetic conditions rather than solely nanoparticle formation. Although optical absorption measurements confirmed nanoparticle formation, comprehensive determination of direct and indirect band-gap energies was beyond the scope of the present study and may provide valuable insight in future investigations.

### SEM-EDX results of ZnO NPs and thin films

**Fig. 3:**
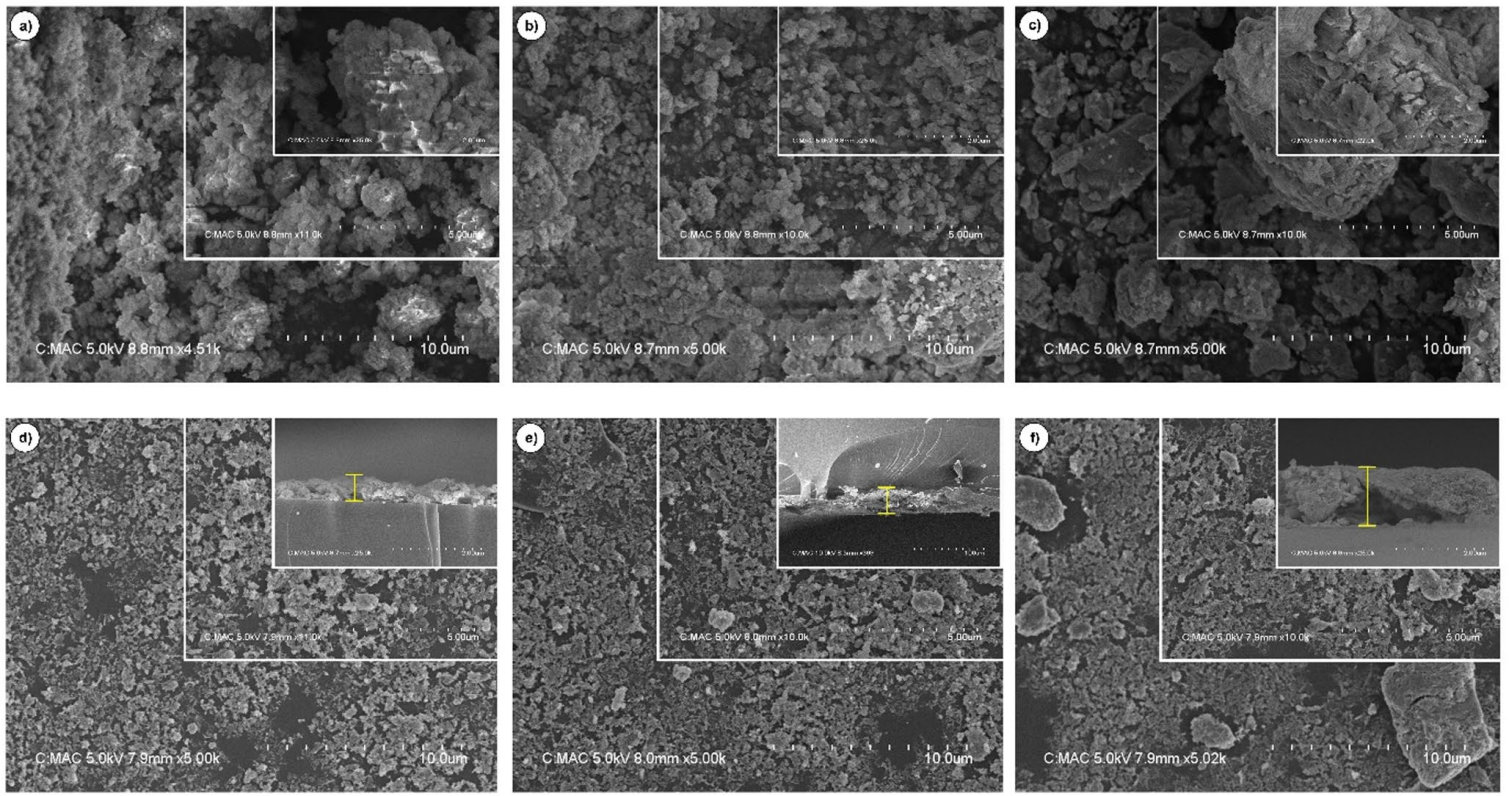
SEM images of ZnO NPs (**a**: ZnO-1 NPs, **b**: ZnO-2 NPs, **c**: ZnO-3 NPs) and thin films (**d**: ZnO-1 thin film, **e**: ZnO-2 thin film, **f**: ZnO-3 thin film). The thicknesses of the thin films are presented in the smallest SEM micrographs provided in the responsible image

The structural and morphological characteristics of the biosynthesized ZnO NPs were assessed through SEM imaging, as depicted in Fig. 3. SEM micrographs revealed that the biosynthesized ZnO NPs predominantly exhibited hexagonal and quasi-spherical morphologies (Fig. 3). A pronounced tendency toward particle aggregation was observed in all samples. The agglomerated structures hindered reliable discrimination of individual particle boundaries, preventing accurate particle-size measurements from SEM images. The observed aggregation behavior may be associated with interparticle interactions and biomolecule-mediated surface effects [90]. Previous studies have reported morphology variations among biosynthesized ZnO nanoparticles depending on microbial origin and synthesis conditions [52, 82]. The differences observed in the present study further support the influence of strain-dependent biosynthetic pathways on nanoparticle organization and morphology.

The surface morphology of ZnO-1, ZnO-2, and ZnO-3 thin films coated on glass substrates is illustrated by SEM micrographs in Fig. 3d and e, and 3f, respectively. The images demonstrate that all films exhibit a compact and homogeneous surface morphology, with no observable cracks or pinholes, confirming the uniformity of the deposition process. No visible detachment or degradation of the films was observed during handling and antibacterial testing, further supporting their mechanical stability and adhesion. Cross-sectional SEM analysis (Fig. 3) further revealed the thickness values of ZnO-1, ZnO-2, and ZnO-3 as 516.09 ± 67.60 nm, 34.37 ± 4.81 μm, and 1.48 ± 0.08 μm, respectively. Such notable variations in thickness strongly suggest that the microbial source employed in NP synthesis exerts a significant influence on the film growth mechanism and structural characteristics. The considerable differences in film thickness suggest that strain-dependent synthesis conditions may affect nanoparticle packing behavior and film growth characteristics, thereby influencing the resulting film architecture. In addition to morphological uniformity, the adhesion quality of the ZnO thin films to the glass substrates was evaluated qualitatively based on surface integrity and structural continuity. The absence of cracks, delamination, or peeling features in SEM micrographs indicates strong interfacial adhesion between the coating and the substrate. Furthermore, the multi-step spin-coating process followed by thermal annealing at 400 °C likely enhanced film densification and bonding strength. These observations collectively suggest that the fabricated ZnO thin films exhibit good adhesion properties, ensuring their stability for potential surface applications.

**Fig. 4:**
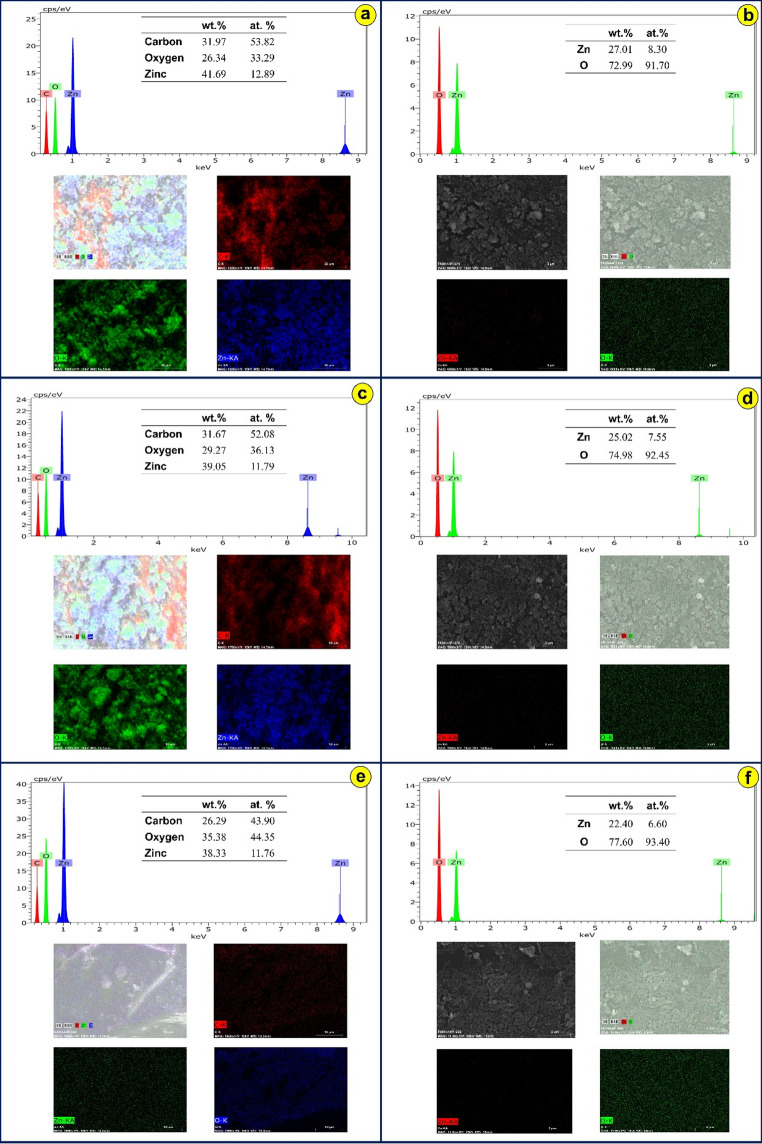
EDX results of ZnO NPs (**a**: ZnO-1 NPs, **c**: ZnO-2 NPs, **e**: ZnO-3 NPs) and thin films (**b**: ZnO-1 thin film, **d**: ZnO-2 thin film, **f**: ZnO-3 thin film)

Elemental composition of the biosynthesized ZnO nanoparticles was verified using EDX spectroscopy. In this study, EDX spectra confirmed the successful synthesis of ZnO NPs obtained using different yeast strains, as shown in Fig. 4. The spectra exhibited a prominent peak around 1 keV corresponding to zinc, along with a medium-intensity peak attributed to oxygen, thereby confirming the formation of ZnO [54]. Quantitative evaluation revealed zinc atomic percentages of 12.89%, 11.79%, and 11.76% for ZnO-1, ZnO-2, and ZnO-3 NPs, respectively, alongside oxygen contents of 33.29%, 36.13%, and 44.35%. Similarly, EDX spectra of ZnO thin films demonstrated zinc and oxygen atomic percentages of 8.30% and 91.70% for ZnO-1, 7.55% and 92.45% for ZnO-2, and 6.60% and 93.40% for ZnO-3. Elemental mapping further illustrated a uniform spatial distribution of zinc and oxygen, with no signs of aggregation, indicating that all ZnO thin films possess a homogeneously dispersed composition. The homogeneous elemental distribution observed across all samples suggests successful formation of ZnO structures with uniform surface composition. The presence of carbon-, phosphorus-, and nitrogen-related signals may be linked to components of the PDB growth medium and residual precursor species remaining from zinc acetate. Notably, the detection of only zinc and oxygen in EDX analysis is indicative of impurity-free ZnO NPs [91]. EDX analysis is primarily used to determine elemental composition, whereas particle size characterization is typically performed using methods tailored to colloidal systems, such as Dynamic Light Scattering (DLS). Nevertheless, for highly aggregated or biomolecule-capped ZnO NPs, dispersion-related limitations may reduce the accuracy of these measurements, necessitating the use of additional characterization methods [92]. DLS-based characterization of ZnO NPs biosynthesized by yeast strains will be addressed in future studies.

The variations observed in the oxygen-to-zinc atomic ratios in the EDX spectra (Fig. 4) suggest differences in surface composition and defect-related characteristics among the biosynthesized ZnO nanostructures. Although stoichiometric ZnO ideally exhibits a Zn: O ratio close to 1:1, deviations from this composition have frequently been reported in green-synthesized systems and are commonly associated with intrinsic defects, surface hydroxylation, and biomolecule-mediated surface modifications [93]. In the present study, the relatively higher oxygen content detected particularly in ZnO-2 and ZnO-3 samples may indicate oxygen-rich surface states arising from adsorbed oxygen species and hydroxyl groups generated during the aqueous biosynthesis process [94]. Such surface enrichment has previously been associated with defect states, including oxygen-related defects and zinc-deficient regions, which can influence the optical, catalytic, and antimicrobial properties of ZnO nanomaterials [95]. Furthermore, differences in Zn/O ratios between nanoparticle powders and thin films may partially arise from variations in film thickness, surface coverage, and signal contributions from the underlying substrate during EDX acquisition [96]. The carbon signals detected in the spectra are likely related to residual biomolecular constituents originating from yeast-derived metabolites and culture medium components, which may remain adsorbed on nanoparticle surfaces following biosynthesis [97]. Collectively, these observations indicate that strain-dependent biosynthetic pathways may influence both the compositional characteristics and surface chemistry of ZnO nanostructures, thereby contributing to differences in their physicochemical behavior and functional performance.

### XRD patterns of green-synthesized ZnO NPs and thin films

**Fig. 5:**
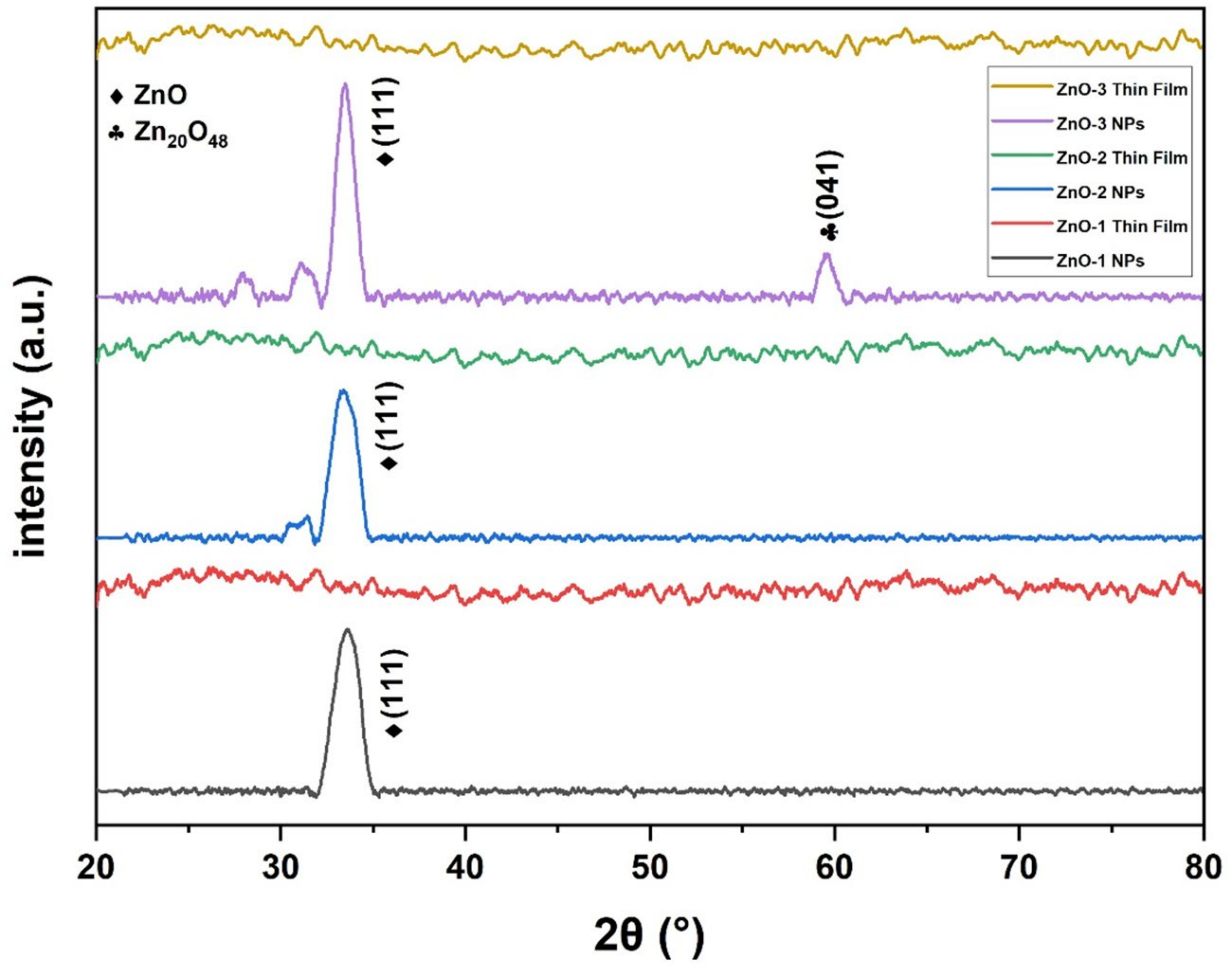
XRD patterns of ZnO NPs and thin film

Crystallographic properties of the biosynthesized ZnO NPs were analyzed using XRD, enabling evaluation of lattice arrangement, orientation behavior, and crystallite dimensions [98]. Although ZnO is commonly known to crystallize in the hexagonal wurtzite structure, several studies have demonstrated that metastable phases such as cubic (zinc blende and rock-salt) and monoclinic forms can be obtained under specific synthesis conditions, particularly in biologically mediated and low-temperature processes [94, 99]. The NPs synthesized using yeast supernatants exhibited distinct diffraction peaks at 2θ values corresponding to the (111) and (041) crystallographic planes, as shown in Fig. 5. These diffraction patterns confirmed the crystalline nature of the NPs and suggested monoclinic (COD code: 4517837; see Supplementary Material, COD_4517837) and cubic (COD code: 1537875; See Supplementary Material, COD_1537875) ZnO phases. Crystallite sizes were calculated using the Scherrer equation. The most intense peaks at 2θ = 33.34° and 33.36° [(111) plane] corresponded to average crystallite sizes of 10.80 nm for both ZnO-1 and ZnO-2 NPs. For *B. anomalus* strain, ZnO-3 NPs, peaks at 2θ = 33.47° and 59.74° [(111) and (041) planes] yielded crystallite sizes of 8.78 nm and 19.40 nm, respectively. The diffraction peaks detected at approximately 2θ = 33° and 59° corresponded to the (111) and (041) crystallographic planes and confirmed the crystalline nature of the biosynthesized ZnO nanostructures. Variations in peak intensity and the number of detectable reflections among samples suggest differences in phase composition and crystallization behavior associated with strain-dependent biosynthesis conditions. The calculated crystallite sizes ranging from 8.78 to 19.40 nm indicate nanoscale crystal formation and may contribute to differences in surface-related properties and functional behavior. Overall, most ZnO NPs displayed a cubic crystallite structure, whereas monoclinic Zn_20_O_48_ crystallite forms were observed in NPs synthesized by *B. anomalus* M3 strain (Table 2). The broadening of XRD peaks reflected nanoscale crystallite size, while the presence of sharp and intense peaks confirmed good crystallinity [100, 101]. These results align with earlier reports in which ZnO NPs synthesized by *S. cerevisiae* and *B. subtilis* exhibited hexagonal wurtzite crystalline structures [52, 54]. Collectively, these findings suggest that NP morphology and crystalline phase are influenced by both the microbial source and synthesis conditions.

**Table 2:** Crystallite sizes of ZnO NPs synthesized by yeast strains calculated by Scherrer equation

NPs	2θ (°)	FWHM	(hkl)	Crystallite type	Crystallite size (nm)
ZnO-1 NPs	33.34	0.768	111	Cubic ZnO	10.80
ZnO-2 NPs	33.36	0.768	111	Cubic ZnO	10.80
ZnO-3 NPs	33.47	0.94464	111	Cubic ZnO	8.78
	59.74	0.47232	041	Monoclinic Zn_20_O_48_	19.40

The absence of distinct diffraction peaks in ZnO thin films suggests a predominantly amorphous or weakly crystalline structure [102, 103]. In spin-coated systems derived from biosynthesized NPs, crystallization is strongly dependent on both thermal treatment and the presence of residual organic species [104]. Although annealing at 400 °C facilitates solvent evaporation and partial densification, this temperature may be insufficient to promote complete crystallization and long-range atomic ordering in ZnO thin films [103, 105]. Residual biomolecules originating from the biosynthesis process may remain associated with nanoparticle surfaces and partially suppress crystal growth and long-range structural ordering [104, 106, 107]. The lack of detectable reflections may further indicate the presence of very small crystalline domains below the detection capability of conventional XRD measurements. In addition, the limited number and broad nature of the diffraction peaks observed in ZnO NPs powders can be explained by nanoscale crystallite size effects and structural heterogeneity. At the nanoscale, peak broadening becomes significant due to reduced crystallite size and increased lattice strain, which may obscure weaker reflections and result in the dominance of only the most intense diffraction planes [108, 109]. In addition, the appearance of only one or two dominant peaks in ZnO nanoparticle powders may result from nanoscale crystallite dimensions, peak broadening effects, structural heterogeneity, and coexistence of multiple crystalline phases [108–110]. Reduced crystallite size and defect-rich structures may suppress weaker reflections, resulting in the predominance of only the most intense crystallographic planes. Structural defects and limited crystallinity commonly observed in biosynthesized ZnO systems may significantly influence their optical characteristics and antimicrobial behavior [89, 111].

### FTIR spectra of green-synthesized ZnO NPs

**Fig. 6:**
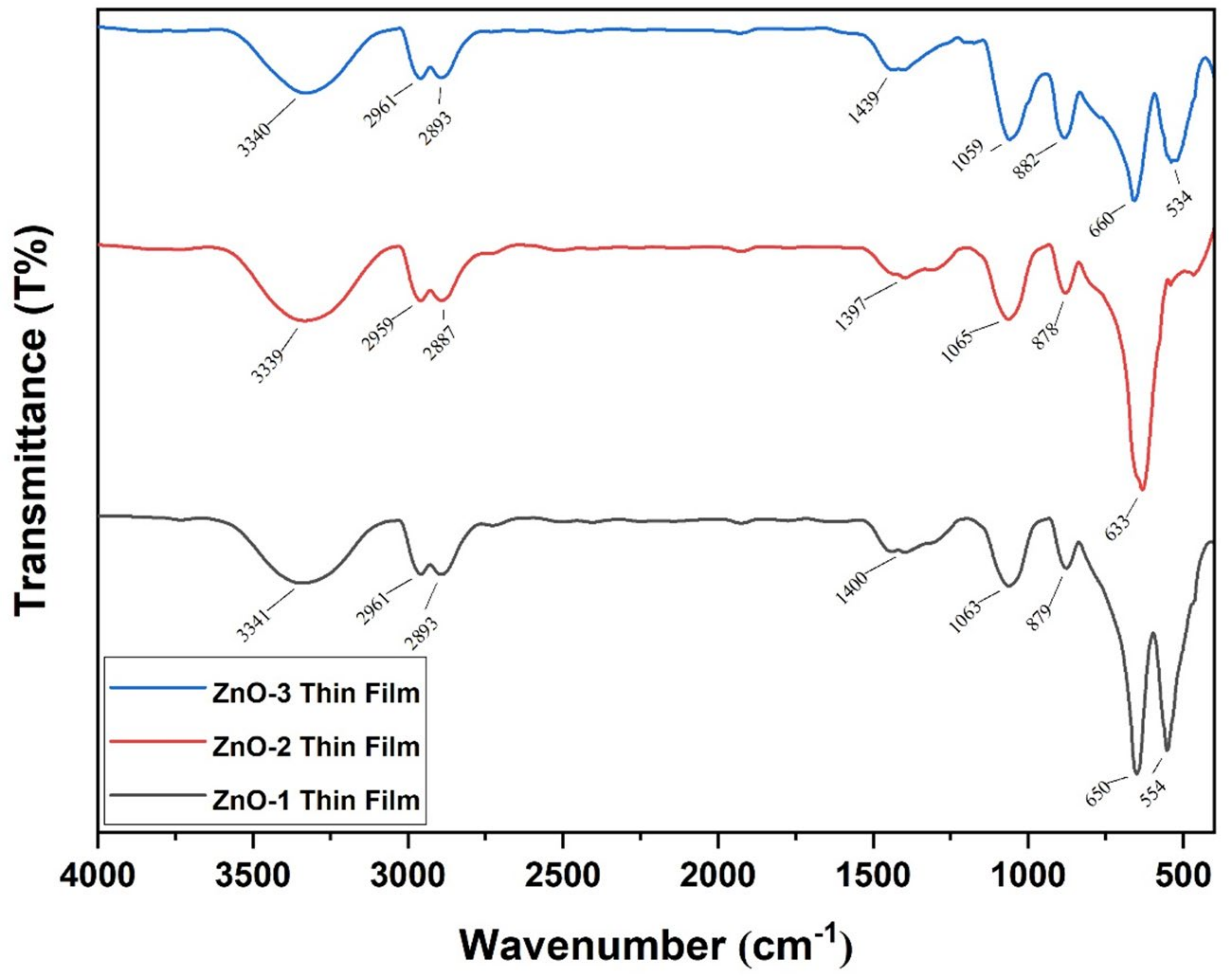
FTIR spectra of ZnO NPs and thin film

FTIR analysis was carried out to determine the functional groups present in the green-synthesized ZnO NPs and to assess the contribution of biomolecule-derived compounds to nanoparticle formation and stabilization. The corresponding spectra are presented in Fig. 6. Characteristic absorption bands were detected in the range of 519–3350 cm^− 1^, indicating the involvement of diverse biomolecules acting as reducing and capping agents during NP synthesis [112, 113]. These findings suggest the binding of unique functional groups from bioactive compounds onto the NP surfaces. The yeast-derived NPs exhibited distinct absorption peaks: ZnO-1 thin film at 3341, 2961, 2893, 1400, 1063, 879, 650 and 554 cm^− 1^; ZnO-2 thin film at 3339, 2959, 2887, 1397, 1065, 878 and 633 cm^− 1^; and ZnO-3 thin film at 3340, 2961, 2893, 1439, 1059, 882, 660 and 534 cm^− 1^ (Fig. 6). The broad peaks observed in the 3319–3350 cm^− 1^ region correspond to –O-H stretching vibrations, indicative of hydroxyl groups from alcohols, phenols, and carboxylic acids [114]. Peaks at 2966–2974 and 2874–2891 cm^− 1^ are attributed to C–H and C–C stretching vibrations of alkanes [114]. The medium-intensity bands observed at approximately 1390 cm^− 1^ and 1560 cm^− 1^ can be attributed to the stretching vibrations of C = O and C = C bonds, respectively [143]. These vibrational features are associated with functional groups present in bioactive compounds such as flavonoids and terpenoids, which are known to act as effective capping and stabilizing agents during the green synthesis of ZnO NPs [107]. Furthermore, variations in the intensity of these bands may indicate partial decomposition or transformation of capping biomolecules during processing or thermal treatment [107]. Importantly, absorption bands in the 519–631 cm^− 1^ region were assigned to Zn–O stretching vibrations, thereby confirming the formation of ZnO NPs [37, 115, 116]. The main FTIR absorption bands, their functional group assignments, and their correlation with previously reported studies are summarized in Table 3.

**Table 3:** FTIR band assignments of biosynthesized ZnO nanoparticles with functional group identification and comparison with literature

Wavenumber (cm^− 1^)	Functional group	Assignment	Interpretation in this study	References
3319–3350	O–H stretching	Hydroxyl groups	Biomolecules (phenols, alcohols, acids) involved in reduction and stabilization	[105–108, 112, 142]
2874–2974	C–H and C–C stretching	Alkanes	Organic residues from yeast-mediated synthesis	
1400–1439	C = O / C = C stretching, C – N stretching/O–H bending	Aromatic biomolecules, Amines/phenolic compounds	Functional groups from bioactive compounds, Flavanoids, Terpenoids	
1057–1078	C–O stretching	Aromatic structures	Contribution of biomoleculer capping agents	
519–631	Zn–O stretching	Metal-oxygen bond	Characteristic ZnO vibration	

* The observed FTIR features are consistent with previously reported biosynthesized ZnO NPs, confirming the role of biomolecules in NPs formation and stabilization

### Water contact angle (WCA) of thin films

**Fig. 7:**
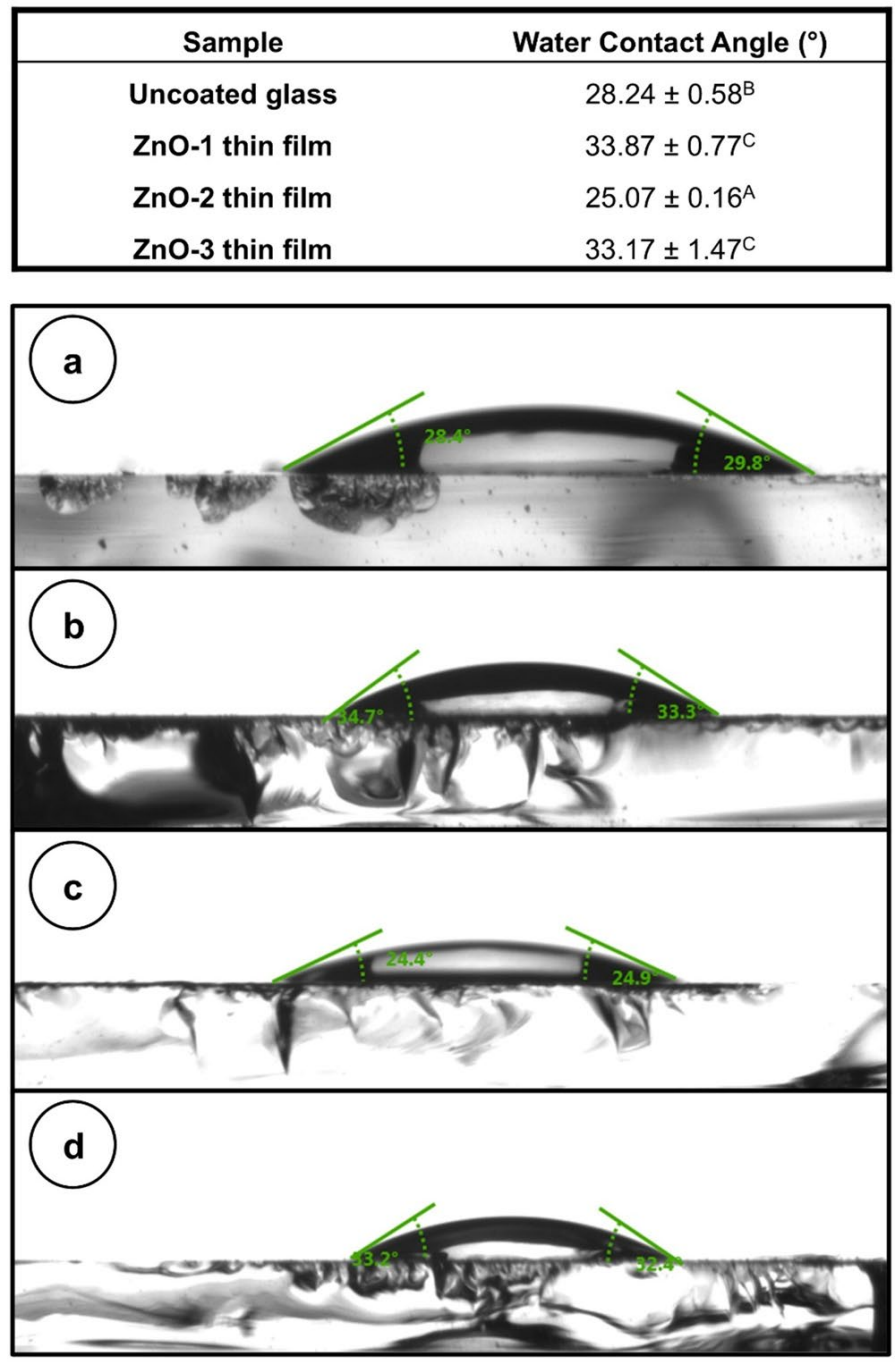
Water contact angle results of thin films (**a**: uncoated glass, **b**: ZnO-1 thin film, **c**: ZnO-2 thin film, **d**: ZnO-3 thin film)

The wettability of ZnO-1, ZnO-2, and ZnO-3 thin film-coated glass substrates was determined using a Krüss DSA100 goniometer. The uncoated glass substrate exhibited a WCA value of 28.24°, whereas ZnO-1, ZnO-2, and ZnO-3 thin film coated substrates showed WCA values of 33.87°, 25.07°, and 33.17°, respectively (Fig. 7). Statistical analysis indicated that ZnO-1 and ZnO-3 thin film coatings resulted in a significant increase in WCA values, while ZnO-2 coatings produced a significant decrease (*p* < 0.05). The WCA values obtained in this study remained below 40°, confirming that all ZnO thin films impart a predominantly hydrophilic character to the glass surfaces. The hydrophilic nature of ZnO-based coatings can be explained by the presence of surface hydroxyl groups and oxygen-related defects, which enhance interactions with water molecules through hydrogen bonding, as also observed in previous studies [55, 117]. The variation in WCA measurements across ZnO thin films suggests that wettability is governed by factors including synthesis conditions, particle size, and surface morphology. The significantly lower water contact angle observed for the ZnO-2 thin film indicates enhanced surface hydrophilicity compared to ZnO-1 and ZnO-3 coatings. This behavior can be attributed to differences in surface chemistry, microstructure, and defect density, all of which are strongly influenced by the biological origin of the NPs [104, 118–120]. The lower WCA value observed for ZnO-2 suggests enhanced surface hydrophilicity, which may be associated with differences in surface chemistry and film morphology. Variations in defect density and surface-associated functional groups may alter surface energy and influence interactions between the coating and water molecules [109, 121]. The enhanced hydrophilic behavior of ZnO-2 may additionally be related to differences in film architecture and thickness, which can modify surface characteristics and wetting behavior. These observations suggest that strain-dependent biosynthesis conditions may influence surface properties through changes in coating structure and composition [69, 122]. Variations in nanoparticle morphology and film density may contribute to differences in surface energy and wettability behavior [123]. Since all WCA values remained below 40°, all ZnO thin films can be classified as hydrophilic. However, the observed differences in antibacterial performance are unlikely to arise solely from minor changes in wettability. Surface chemistry, defect characteristics, and nanoscale morphology likely play more important roles in determining functional behavior, consistent with previous findings for ZnO coatings [120].

### Antibacterial activity of green-synthesized ZnO NPs and thin films

The antibacterial performance of ZnO NPs synthesized from yeast strain-derived supernatants was investigated using *E. coli* O157:H7 and *S. aureus* as representative Gram-negative and Gram-positive bacterial strains, respectively. All ZnO NPs demonstrated significant inhibitory activity, with inhibition zones ranging from 13.00 ± 2.12 to 22.00 ± 0.71 mm, as shown in Table 4. The most pronounced effect was observed for ZnO NPs synthesized by *Z. bailii* strain M1. To better contextualize these findings, a comparison with previously reported studies reveals that the antibacterial performance of the present ZnO NPs is consistent with, and in some cases comparable to, other biosynthesized systems. For instance, ZnO NPs synthesized using *S. cerevisiae* exhibited inhibition zones of approximately 23.1 mm against *S. aureus* and 17.0 mm against *E. coli*, demonstrating a similar trend of stronger activity against Gram-positive bacteria [52, 124]. This behavior is widely reported and is generally attributed to structural differences in bacterial cell walls, where the thicker peptidoglycan layer in Gram-positive bacteria facilitates stronger interactions with ZnO NPs. Similar antibacterial performance ranges (⁓13–22 mm) have been reported in yeast-mediated ZnO systems, depending on synthesis conditions and NPs characteristics such as size, crystallinity, and defect density [84]. Moreover, earlier studies have demonstrated that ZnO NPs synthesized via microbial routes (such as *P. fermentans*) exhibit variable antibacterial activity depending on the target microorganism and synthesis pathway, highlighting the critical role of biological origin in determining antimicrobial efficiency [125]. In addition, it is well established that smaller nanoparticle size and higher surface area-to-volume ratio enhance antibacterial activity by increasing nanoparticle-cell interactions and promoting ROS generation [126]. The antibacterial mechanism of ZnO NPs is further governed by Zn^2+^ ion release, ROS production, and direct membrane disruption, all of which are strongly dependent on NP physicochemical properties [127, 128]. Taken together, these comparisons demonstrate that the antibacterial performance observed in this study falls well within the range reported in the literature for biosynthesized ZnO NPs. The variations between samples can be attributed to differences in synthesis conditions, NP morphology, and surface chemistry, confirming that the green synthesis route plays a critical role in tailoring antibacterial activity [54].

**Table 4:** Antibacterial activity of ZnO NPs synthesized by yeast strains

Sample	Inhibition diameter of ZnO NPs (mm)*			MIC values of ZnO NPs (mg/mL)		MBC values of ZnO NPs (mg/mL)	
	*E. coli* O157:H7		*S. aureus*	*E. coli* O157:H7	*S. aureus*	*E. coli*i O157:H7	*S. aureus*
ZnO-1 NPs	21.00 ± 0.00		22.00 ± 0.71	0.313 ± 0.00	0.313 ± 0.00	0.625 ± 0.00	0.625 ± 0.00
ZnO-2 NPs	18.00 ± 0.00		19.00 ± 0.71	0.313 ± 0.00	0.625 ± 0.00	0.313 ± 0.00	0.625 ± 0.00
ZnO-3 NPs	13.00 ± 2.12		20.00 ± 0.00	0.625 ± 0.00	0.625 ± 0.00	0.625 ± 0.00	1.25 ± 0.00

*Well diameter was not included

The MIC values of ZnO NPs were 0.313–0.625 mg/mL for both pathogenic cultures. Consistent with inhibition zone results, ZnO-1 NPs exhibited the greatest antibacterial efficacy. Bactericidal activity was observed at concentrations between 0.313 and 1.25 mg/mL (Table 4). Interestingly, ZnO-2 NPs exhibited identical MIC and MBC values for both pathogens (0.313 mg/mL for *E. coli* O157:H7 and 0.625 mg/mL for *S. aureus*). Similarly, identical values were reported for ZnO-3 NPs against *E. coli* O157:H7 (0.625 mg/mL). Comparative studies underline the importance of the synthesis source: Elsilk et al. [129] observed activity of *Enterobacter*-mediated ZnO NPs against *E. coli*, *S. typhimurium*, *Klebsiella pneumoniae*, and *C. albicans*, with MIC values of 0.4, 0.9, 1.0, and 1.5 mg/mL, respectively.

**Fig. 8:**
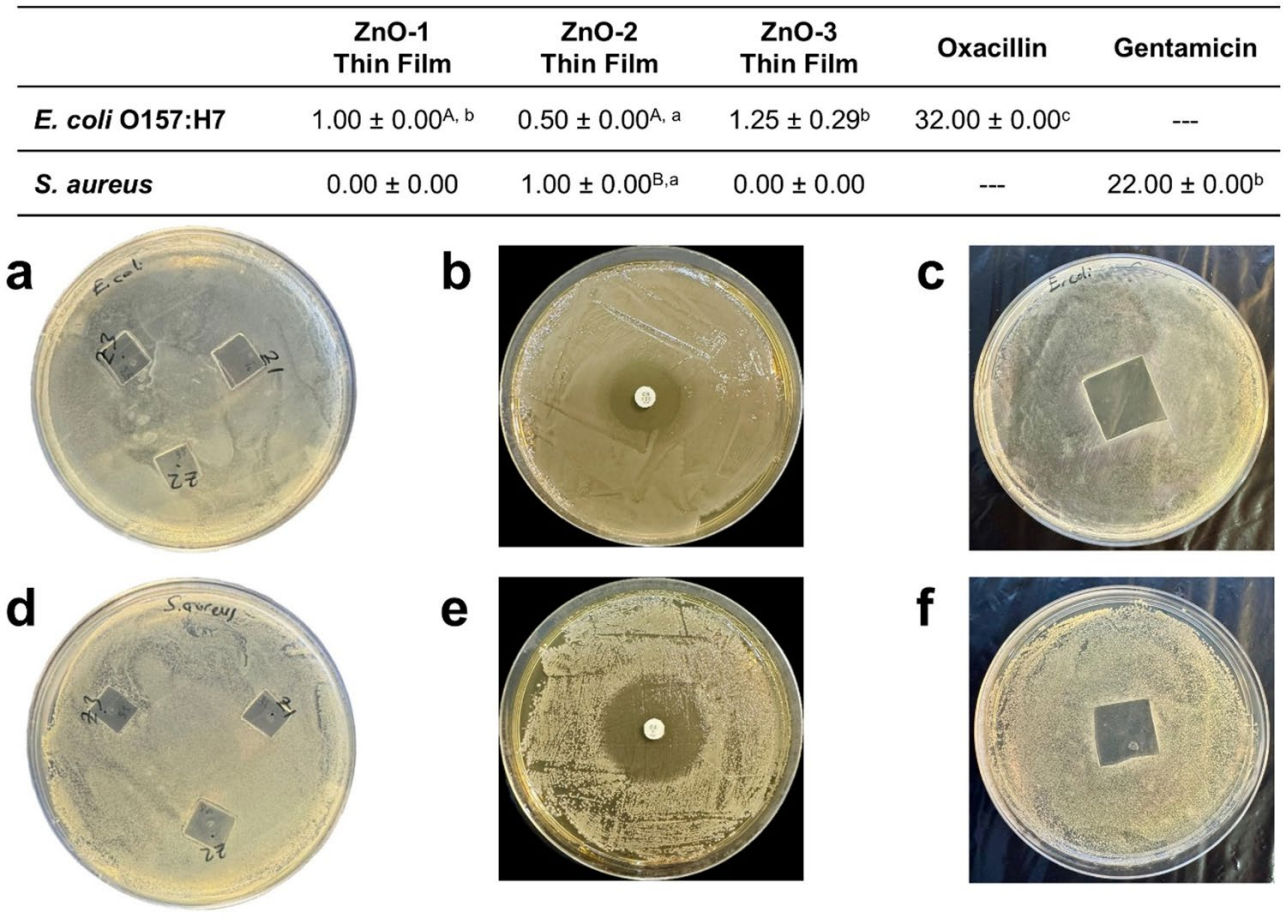
Antibacterial activity of ZnO thin film-coated glass substrates evaluated by the modified Kirby–Bauer disk diffusion method against *Escherichia coli* O157:H7 (a-c) and *Staphylococcus aureus* ATCC 6538P (d-f). (a, d) ZnO-1, ZnO-2, and ZnO-3 thin films; (b, e) positive control antibiotic discs (gentamicin for *E. coli* and oxacillin for *S. aureus*); and (c, f) negative control represented by uncoated glass substrates. The corresponding inhibition zone diameters (mm) are presented in the table above. Data are expressed as mean ± standard deviation of triplicate measurements. Different lowercase letters (a, b) within the same row and different uppercase letters (A, B) within the same column indicate statistically significant differences (*p* < 0.05) between mean values

The antibacterial activity of ZnO NP-coated glass substrates was assessed against *E. coli* O157:H7 and *S. aureus* using a modified Kirby-Bauer method (Fig. 8). Among the samples, ZnO-3 thin films demonstrated the largest inhibition zone (1.25 mm) against *E. coli* O157:H7, whereas ZnO-1 and ZnO-3 films showed no detectable inhibition (0.00 mm) against *S. aureus*. In contrast, ZnO-2 thin films produced measurable inhibition zones of 0.50 mm and 1.00 mm against *E. coli* and *S. aureus*, respectively.

Direct contact antibacterial tests were performed by applying approximately 10^4^ CFU/cm^2^ of each bacterial strain onto 1 × 1 cm ZnO NP-coated and uncoated glass surfaces. Bacterial viability was assessed at the initial time point (t = 0 h) and after 2 h of incubation at room temperature (t = 2 h). The inhibition rate was determined according to the following formula:

$$\% \,{\mathrm{inhibition}} = \left[ {\left( {{\mathrm{N}}_{{{\mathrm{t}}:0}} - {\mathrm{N}}_{{{\mathrm{t}}:{\mathrm{2}}}} } \right)/{\mathrm{N}}_{{{\mathrm{t}}:0}} } \right] \times {\mathrm{1}}00,$$

where N_t:0_ represents the initial inoculum (CFU/cm^2^) and N_t:2_ denotes the surviving microbial load after 2-h of contact with ZnO NPs-coated surfaces. The results are summarized in Table 5. As shown, both pathogenic cultures, *E. coli* O157:H7 and *S.* aureus, were completely inhibited by all developed ZnO thin films. The apparent discrepancy between the WCA results and antibacterial performance, particularly in the case of the ZnO-2 thin film, highlights that surface wettability alone is not the dominant factor governing antibacterial activity. While lower contact angle values indicate increased hydrophilicity and may facilitate initial bacterial adhesion, the overall antibacterial efficacy of ZnO-based coatings is primarily dictated by physicochemical factors such as surface defect density, Zn^2+^ ion release, ROS generation, and the accessibility of active surface sites [94, 130]. In the present study, despite exhibiting the lowest contact angle, the ZnO-2 thin film demonstrated comparatively lower inhibition efficiency, which can be attributed to its distinct structural characteristics. SEM analysis revealed that the thin films possess compact and relatively uniform morphologies with varying thicknesses, and the significantly greater thickness of the ZnO-2 film is likely to reduce the effective surface-to-volume ratio and limit the exposure of catalytically active sites responsible for antibacterial action [131, 132]. In addition, differences in NP packing density and surface coverage may hinder direct contact between bacterial cells and reactive ZnO surfaces, thereby decreasing antibacterial effectiveness [133]. Furthermore, FTIR analysis confirmed the presence of surface hydroxyl groups and biomolecule-derived functional species associated with the green synthesis route, which can influence surface energy, NP stabilization, and interfacial interactions with bacterial membranes. Although such functional groups contribute to increased hydrophilicity, they may also partially passivate the ZnO surface and reduce the availability of active sites involved in antibacterial processes. In parallel, UV-Vis spectra revealed characteristic ZnO absorption behavior with variations in transmittance among the samples, suggesting differences in defect states and electronic structure. Since defect-rich ZnO surfaces, particularly those containing oxygen vacancies, are known to enhance ROS generation, variations in defect density may directly affect antibacterial efficiency [134]. Therefore, the results indicate that although hydrophilicity can influence surface-bacteria interactions, it does not necessarily correlate directly with antibacterial efficiency. Instead, antibacterial activity arises from a complex interplay of structural, optical, and surface chemical factors, which must be considered collectively when interpreting the performance of ZnO thin films.

**Table 5:** The viable cell counts of pathogenic cultures following direct contact time at 0 (t: 0) and 2nd (t: 2) hours on the glass substrates coated with the ZnO NPs

Thin films	*E. coli* O157:H7 (log CFU/cm^2^)		*S. aureus* (log CFU/cm^2^)	
	t: 0	t: 2	t: 0	t: 2
Uncoated glass	4.35 ± 0.15^A, a^	4.27 ± 0.06^a^	4.06 ± 0.19^A, a^	3.82 ± 0.36^a^
ZnO-1 thin film	4.21 ± 0.36^A^	< 0.7	3.89 ± 0.35^A^	< 0.7
ZnO-2 thin film	4.07 ± 0.18^A^	< 0.7	4.21 ± 0.34^A^	< 0.7
ZnO-3 thin film	4.11 ± 0.03^A^	< 0.7	4.12 ± 0.23^A^	< 0.7

*Mean values in the same row that are not followed by the same lower-case letter (a-b) are significantly different (*p* < 0.05). Mean values in the same column that are not followed by the same capital letter (A-D) are significantly different (*p* < 0.05). The limit of detection is 0.7 log CFU/cm^2^ for direct contact analysis of ZnO thin films

The antibacterial activity of the biosynthesized ZnO nanostructures may involve multiple mechanisms, including membrane interactions, Zn^2+^ ion release, and ROS-associated oxidative effects, as proposed in previous studies [13, 135]. Previous reports suggest that ZnO NPs may interact with bacterial membranes, potentially affecting membrane integrity and cellular functions [135]. In addition, Zn²⁺ ion release has been proposed as a contributing factor affecting intracellular processes and bacterial viability [135]. Recent studies have likewise shown that soluble Zn^2+^ can be a major contributor under some media conditions, whereas membrane injury and NPs surface reactivity remain critical determinants of the overall response, indicating that the dominant pathway is environment- and material-dependent [133, 136]. Another frequently proposed mechanism involves ROS-mediated oxidative effects. ZnO nanostructures have been reported to generate ROS under certain conditions, including superoxide radicals, hydroxyl radicals, and hydrogen peroxide, which may contribute to oxidative stress-related antibacterial effects [13]. The diminished antibacterial activity reported in the presence of radical scavengers such as glutathione, vitamin E, and mannitol further supports the central role of ROS in ZnO-mediated bactericidal action. Importantly, ROS generation is closely related to surface defect chemistry, particularly oxygen vacancies and other defect-rich surface states, which enhance redox activity and can amplify antimicrobial performance [94, 137]. The present findings may be interpreted within the framework of these proposed antibacterial mechanisms. The larger inhibition zones observed for ZnO NPs suspensions relative to thin films suggest that greater accessible surface area and closer NP-cell contact promote stronger antibacterial action. Likewise, the lower inhibition efficiency of the ZnO-2 thin film, despite its lower WCA, indicates that hydrophilicity alone does not determine antibacterial performance; rather, film thickness, packing density, and the accessibility of reactive ZnO surface sites appear to be more decisive. In addition, the FTIR spectra confirmed the presence of hydroxyl-containing and biomolecule-derived surface functionalities, while the UV-Vis data suggested differences in defect-related optical behavior among the samples, both of which may influence ROS formation and interfacial reactivity. Together with SEM observations and physicochemical characterization results, these findings suggest that antibacterial performance may be influenced by a combination of surface interactions, ionic effects, and oxidative processes associated with the structural characteristics of biosynthesized ZnO nanostructures [13, 133, 136]. In agreement with recent studies, the antibacterial efficiency of ZnO NPs is strongly dependent on particle size, surface characteristics, and synthesis route, which directly influence NP-cell interactions and ROS generation [138]. In addition, green-synthesized ZnO NPs exhibit enhanced biological activity due to biomolecule-derived capping agents, which improve stability and facilitate interaction with bacterial membranes [139]. Furthermore, ZnO-based nanostructures have shown strong potential for applications such as antimicrobial coatings, wound healing systems, and controlled ion-release platforms [140]. Overall, the antibacterial findings suggest a close relationship between the physicochemical characteristics of the synthesized ZnO nanostructures and their biological performance. Variations in crystallite size identified by XRD, aggregation behavior observed in SEM images, surface-related functional groups detected by FTIR, optical differences revealed by UV-Vis analysis, and wettability characteristics determined by WCA measurements collectively indicate that antibacterial activity is governed by multiple interconnected parameters rather than a single dominant factor. These observations support a structure-property-activity relationship in which nanoscale structural features and surface characteristics jointly influence antibacterial effectiveness. Although the antibacterial performance observed in this study is notable, certain limitations should be considered. In agar-based diffusion assays, the restricted mobility of ZnO NPs may affect inhibition zone formation and may not fully represent their intrinsic antibacterial activity. Moreover, for thin film systems, antibacterial performance is predominantly governed by direct surface contact, where variations in film uniformity and surface coverage can influence interactions with bacterial cells. In addition, differences in bacterial cell wall structure and susceptibility may further contribute to variability in the observed antibacterial responses. However, direct mechanistic analyses such as ROS quantification, membrane integrity evaluation, or related biochemical investigations were beyond the scope of the present study. Therefore, the proposed mechanisms should be considered literature-supported interpretations and warrant further investigation in future studies.

Despite extensive studies on ZnO nanomaterials, limited attention has been given to the combined evaluation of biosynthesized ZnO NPs and their corresponding thin film forms in relation to their structure-property-activity relationships. In particular, the influence of green synthesis-derived surface characteristics on both wettability and antibacterial performance remains insufficiently understood. The present study contributes to addressing this gap by providing a systematic comparison of NP and thin film systems and linking their physicochemical characteristics with antibacterial behavior. Future research should focus on optimizing synthesis parameters to achieve better control over particle size, morphology, and defect density, which are recognized as important factors influencing antibacterial performance. In addition, surface modification and functionalization strategies may further enhance the stability and effectiveness of ZnO NPs. Comprehensive investigations on long-term stability, cytocompatibility, and performance under real-world application conditions are also necessary to advance the practical implementation of these materials. Furthermore, tailoring the interactions between biomolecule-derived capping agents and ZnO surfaces may offer new opportunities to fine-tune functional properties.

## Conclusion

A green synthesis approach was successfully employed for the production of ZnO NPs using extracellular extracts derived from *Z. bailii* M1 and *B. anomalus* M2 and M3, followed by fabrication of thin-film coatings through spin coating. Physicochemical characterization confirmed successful nanoparticle synthesis and coating formation, with SEM revealing aggregated nanoparticle morphologies and homogeneous films of varying thicknesses. XRD analysis demonstrated nanoscale crystallinity in ZnO NPs, whereas thin films exhibited predominantly amorphous characteristics. FTIR analysis further confirmed Zn–O bonding and the presence of biomolecule-associated surface functional groups originating from the biosynthesis route. WCA analysis demonstrated hydrophilic surface behavior for all coatings; however, antibacterial performance did not directly correlate with wettability. In particular, the ZnO-2 thin film exhibited enhanced hydrophilicity without showing the highest antibacterial activity, suggesting that antibacterial behavior is governed by multiple physicochemical factors rather than wettability alone. Variations in crystallite size, morphology, film architecture, and surface characteristics collectively indicate a structure-property-activity relationship influencing antibacterial performance. ZnO NPs and their corresponding thin films demonstrated notable antibacterial activity against *E. coli* O157:H7 and *S. aureus*. Based on physicochemical findings and previous literature, antibacterial activity may be associated with multiple pathways involving surface interactions, Zn^2+^ ion release, and oxidative processes; however, direct mechanistic investigations remain necessary for definitive confirmation. Overall, the findings highlight the potential of biosynthesized ZnO nanostructures as sustainable antimicrobial platforms and provide insight into the role of physicochemical characteristics in determining functional performance. Future investigations should focus on mechanistic validation, optimization of synthesis parameters, and evaluation under practical application conditions.

## Supplementary Information

Below is the link to the electronic supplementary material.

Supplementary Material 1

Supplementary Material 2

## Data Availability

Data will be available on request.

## Declarations

### Generative AI and AI-assisted technologies in the writing process

During the preparation of this work the authors used GPT5.1 in order to improve the readability and language. After using this tool/service, the authors reviewed and edited the contents as needed and take full responsibility for the content of the publication.
