# Supplementary material for: Eco-friendly synthesis of ZnO nanostructures from yeast strains isolated from kombucha and beetroot kwass for antimicrobial thin film applications
Source: Bioprocess Biosyst Eng. 2026 Jun 25;49(7):1885–907. doi: 10.1007/s00449-026-03372-0 (PMC13379424; doi:10.1007/s00449-026-03372-0)
Supplement: Supplementary file 1 — Supplementary Material 1 [file 449_2026_3372_MOESM1_ESM.pdf]

Name and formula

Reference code: 96-153-7876

Compound name: Zn O
Common name: Zn O

Chemical formula: $O_{4.00}Zn_{4.00}$

Crystallographic parameters

Crystal system: Cubic
Space group: F -4 3 m
Space group number: 216

a (Å): 4,6290
b (Å): 4,6290
c (Å): 4,6290
Alpha (°): 90,0000
Beta (°): 90,0000
Gamma (°): 90,0000

Calculated density (g/cm^3): 5,45
Volume of cell (10^6 pm^3): 99,19

RIR: 9,15

Subfiles and Quality

Subfiles: User Inorganic
Quality: None ( )

Comments

Creation Date: 7.05.2024 03:29:37
Modification Date: 7.05.2024 03:29:37
Publication title: The structure of thin films of certain metallic oxides
COD database code: 1537875

References

Structure: Bragg, W., *Transactions of the Faraday Society*, **28**, 522 - 522, (1932)

Peak list

No.	h	k	l	d [A]	2Theta[deg]	I [%]
1	1	1	1	2,67255	33,504	100,0
2	0	2	0	2,31450	38,879	26,2
3	0	2	2	1,63660	56,156	41,3
4	1	3	1	1,39570	66,996	31,3
5	2	2	2	1,33628	70,403	5,4
6	0	4	0	1,15725	83,461	5,1
