# Supplementary material for: Eco-friendly synthesis of ZnO nanostructures from yeast strains isolated from kombucha and beetroot kwass for antimicrobial thin film applications
Source: Bioprocess Biosyst Eng. 2026 Jun 25;49(7):1885–907. doi: 10.1007/s00449-026-03372-0 (PMC13379424; doi:10.1007/s00449-026-03372-0)
Supplement: Supplementary file 2 — Supplementary Material 2 [file 449_2026_3372_MOESM2_ESM.pdf]

Name and formula

Reference code: 96-451-7838

Compound name: Zinc Hydroxide Dihydrate
Common name: Zinc Hydroxide Dihydrate

Chemical formula: $Zn_{20.00}O_{48.00}$

Crystallographic parameters

Crystal system: Monoclinic
Space group: C 1 2/c 1
Space group number: 15

a (Å): 15,3420
b (Å): 6,2440
c (Å): 10,9890
Alpha (°): 90,0000
Beta (°): 100,8600
Gamma (°): 90,0000

Calculated density (g/cm^3): 3,33
Volume of cell (10^6 pm^3): 1033,84

RIR: 4,40

Subfiles and Quality

Subfiles: User Inorganic
Quality: None ( )

Comments

Creation Date: 5.05.2024 21:09:15
Modification Date: 5.05.2024 21:09:15
Crystal color: white
Crystal description: plate like nano powder
Publication title: Layered Zinc Hydroxide Dihydrate, Zn₅(OH)₁₀&x00B7;2H₂O, from Hydrothermal Conversion of e-Zn(OH)₂ at Gigapascal Pressures and its Transformation to Nanocrystalline ZnO.
COD database code: 4517837

References

Structure: Gordeeva, Alisa, Hsu, Ying-Jui, Jenei, Istvan Z., Brant Carvalho, Paulo H. B., Simak, Sergei I., Andersson, Ove, H'aussermann, Ulrich , *ACS omega*, **5**, 17617 - 17627, (2020)

Peak list

No.	h	k	l	d [A]	2Theta[deg]	I [%]
1	2	0	0	7,53362	11,737	100,0
2	1	1	0	5,76830	15,348	1,9
3	0	0	2	5,39610	16,414	2,8

4	-1	1	1	5,24701	16,884	0,1
5	1	1	1	4,94123	17,937	0,1
6	-2	0	2	4,83972	18,317	0,0
7	-1	1	2	4,09058	21,708	2,9
8	2	0	2	4,04120	21,977	6,1
9	3	1	0	3,91351	22,703	19,5
10	-3	1	1	3,86545	22,990	0,3
11	1	1	2	3,80605	23,353	0,6
12	4	0	0	3,76681	23,600	3,0
13	3	1	1	3,51732	25,301	0,0
14	-3	1	2	3,41529	26,070	5,1
15	-4	0	2	3,40439	26,155	8,8
16	-1	1	3	3,15638	28,251	0,0
17	0	2	0	3,12200	28,569	3,2
18	0	2	1	2,99903	29,766	0,1
19	3	1	2	2,96773	30,088	11,8
20	1	1	3	2,95814	30,188	0,0
21	2	2	0	2,88415	30,981	3,7
22	-3	1	3	2,86641	31,178	0,0
23	4	0	2	2,84717	31,394	6,1
24	-2	2	1	2,83789	31,499	0,3
25	-5	1	1	2,74442	32,601	0,0
26	2	2	1	2,73755	32,686	0,1
27	5	1	0	2,71392	32,978	8,3
28	-2	0	4	2,70712	33,063	7,2
29	0	2	2	2,70231	33,124	14,4
30	0	0	4	2,69805	33,178	6,9
31	-2	2	2	2,62350	34,149	19,9
32	-5	1	2	2,60873	34,348	8,5
33	5	1	1	2,53231	35,419	0,0
34	-1	1	4	2,51452	35,678	3,2
35	6	0	0	2,51121	35,726	0,1
36	3	1	3	2,47369	36,287	0,0
37	2	2	2	2,47061	36,334	0,1
38	-6	0	2	2,46101	36,480	1,8
39	-4	0	4	2,41986	37,123	0,1
40	-4	2	1	2,40848	37,305	0,0
41	4	2	0	2,40372	37,382	3,1
42	2	0	4	2,40055	37,433	4,8
43	-3	1	4	2,39143	37,581	1,0
44	1	1	4	2,37896	37,785	2,0
45	-5	1	3	2,36835	37,961	0,0
46	0	2	3	2,35788	38,136	0,0
47	-2	2	3	2,33390	38,543	0,2
48	-4	2	2	2,30095	39,118	6,4
49	4	2	1	2,28856	39,338	0,1
50	5	1	2	2,27456	39,590	2,1
51	2	2	3	2,17498	41,484	0,1
52	6	0	2	2,12849	42,434	0,2
53	-4	2	3	2,11981	42,616	0,1
54	4	2	2	2,10372	42,958	0,1
55	-5	1	4	2,09982	43,042	2,1
56	3	1	4	2,08300	43,407	2,2
57	-1	1	5	2,07118	43,667	0,0
58	-7	1	1	2,06790	43,740	0,0
59	1	3	0	2,06176	43,877	0,1
60	-2	2	4	2,04529	44,249	0,5
61	0	2	4	2,04137	44,339	0,0
62	-6	0	4	2,03983	44,374	0,4
63	-1	3	1	2,03482	44,489	0,0

64	-7	1	2	2,02698	44,670	0,5
65	4	0	4	2,02060	44,819	1,3
66	-3	1	5	2,01957	44,843	0,0
67	1	3	1	2,01558	44,937	0,0
68	5	1	3	2,00885	45,096	0,0
69	-6	2	1	1,97699	45,863	0,0
70	1	1	5	1,97532	45,904	0,1
71	6	2	0	1,95676	46,365	1,5
72	-1	3	2	1,94273	46,720	0,5
73	7	1	1	1,93784	46,844	0,0
74	-6	2	2	1,93272	46,976	3,1
75	-7	1	3	1,92416	47,198	0,0
76	3	3	0	1,92277	47,234	1,8
77	-3	3	1	1,91699	47,385	0,0
78	-4	2	4	1,91260	47,500	1,8
79	1	3	2	1,90962	47,579	0,0
80	2	2	4	1,90303	47,754	1,2
81	4	2	3	1,89608	47,940	0,0
82	-8	0	2	1,89260	48,034	0,1
83	8	0	0	1,88340	48,283	2,7
84	6	2	1	1,87758	48,442	0,0
85	3	3	1	1,86981	48,657	0,0
86	-3	3	2	1,85400	49,099	0,7
87	-5	1	5	1,84906	49,239	0,0
88	-6	2	3	1,83577	49,619	0,0
89	-2	0	6	1,82910	49,813	0,1
90	-1	3	3	1,80903	50,404	0,0
91	7	1	2	1,80107	50,642	0,1
92	0	0	6	1,79870	50,714	0,0
93	-2	2	5	1,79118	50,942	0,0
94	-7	1	4	1,78465	51,142	1,2
95	3	1	5	1,78260	51,205	0,0
96	0	2	5	1,77543	51,426	0,0
97	3	3	2	1,77127	51,556	1,1
98	5	1	4	1,76918	51,622	0,8
99	6	2	2	1,75866	51,953	3,2
100	-4	0	6	1,75696	52,007	0,1
101	-1	1	6	1,75347	52,118	0,1
102	-3	3	3	1,74900	52,262	0,0
103	-3	1	6	1,73378	52,756	0,0
104	-5	3	1	1,72014	53,207	0,0
105	5	3	0	1,71256	53,461	1,5
106	-4	2	5	1,71153	53,496	0,0
107	-6	2	4	1,70765	53,627	1,1
108	-8	0	4	1,70219	53,813	1,6
109	4	2	4	1,69632	54,014	1,3
110	6	0	4	1,68654	54,353	2,1
111	-5	3	2	1,68518	54,401	1,2
112	1	1	6	1,68300	54,477	0,8
113	8	0	2	1,68232	54,501	0,1
114	2	0	6	1,67951	54,600	0,6
115	2	2	5	1,67123	54,893	0,0
116	5	3	1	1,66404	55,150	0,0
117	-1	3	4	1,65896	55,333	0,3
118	7	1	3	1,64974	55,669	0,0
119	3	3	3	1,64708	55,767	0,0
120	-9	1	1	1,64360	55,895	0,0
121	-8	2	1	1,63396	56,254	0,0
122	-9	1	2	1,63283	56,297	0,1
123	-5	1	6	1,63225	56,319	0,8

124	-3	3	4	1,62210	56,703	0,2
125	6	2	3	1,62188	56,711	0,0
126	-8	2	2	1,61844	56,843	2,6
127	1	3	4	1,61819	56,852	0,3
128	9	1	0	1,61702	56,897	0,2
129	-5	3	3	1,61484	56,981	0,0
130	-6	0	6	1,61324	57,043	0,5
131	8	2	0	1,61268	57,065	0,2
132	-9	1	3	1,58681	58,083	0,0
133	5	3	2	1,58415	58,189	0,4
134	-2	2	6	1,57819	58,430	5,0
135	-8	2	3	1,56909	58,802	0,0
136	-6	2	5	1,56884	58,813	0,0
137	5	1	5	1,56489	58,976	0,0
138	0	4	0	1,56100	59,137	2,5
139	0	2	6	1,55854	59,240	2,2
140	9	1	1	1,55816	59,256	0,0
141	3	1	6	1,55026	59,588	1,0
142	0	4	1	1,54492	59,815	0,0
143	-4	2	6	1,53115	60,409	1,0
144	2	4	0	1,52853	60,523	1,3
145	-10	0	2	1,52768	60,560	0,0
146	-5	3	4	1,52147	60,834	0,7
147	4	2	5	1,51783	60,995	0,0
148	-1	1	7	1,51700	61,032	0,0
149	4	0	6	1,51588	61,082	0,1
150	3	3	4	1,51503	61,120	0,5
151	-9	1	4	1,51378	61,176	0,2
152	-3	1	7	1,51215	61,249	0,0
153	-1	3	5	1,51047	61,324	0,0
154	-7	3	1	1,50919	61,382	0,0
155	10	0	0	1,50672	61,493	0,4
156	2	4	1	1,50546	61,550	0,0
157	7	1	4	1,50118	61,745	0,1
158	0	4	2	1,49952	61,821	0,1
159	7	3	0	1,49624	61,971	0,0
160	-8	2	4	1,49449	62,052	0,1
161	-7	3	2	1,49307	62,118	0,1
162	-3	3	5	1,49010	62,255	0,0
163	5	3	3	1,48577	62,457	0,0
164	-2	4	2	1,48564	62,463	0,0
165	-7	1	6	1,48536	62,476	0,5
166	6	2	4	1,48387	62,546	0,0
167	8	2	2	1,48099	62,681	3,4
168	2	2	6	1,47907	62,772	0,1
169	9	1	2	1,47683	62,878	0,0
170	1	3	5	1,47205	63,105	0,0
171	1	1	7	1,46336	63,524	0,0
172	7	3	1	1,45635	63,866	0,0
173	2	4	2	1,45614	63,876	0,3
174	-7	3	3	1,45051	64,153	0,0
175	-5	1	7	1,45039	64,159	0,0
176	-8	0	6	1,44372	64,491	0,1
177	-4	4	1	1,44310	64,523	0,0
178	4	4	0	1,44208	64,574	0,0
179	-10	0	4	1,43567	64,897	1,6
180	-6	2	6	1,43321	65,023	0,0
181	0	4	3	1,43200	65,084	0,0
182	-2	4	3	1,42657	65,363	0,0
183	-9	1	5	1,42450	65,470	0,0

184	8	0	4	1,42358	65,517	0,4
185	-4	4	2	1,41895	65,758	0,4
186	-5	3	5	1,41751	65,833	0,0
187	4	4	1	1,41603	65,911	0,0
188	-8	2	5	1,40517	66,486	0,0
189	-2	2	7	1,40237	66,636	0,0
190	7	3	2	1,39554	67,005	0,0
191	5	1	6	1,39431	67,072	0,0
192	8	2	3	1,39023	67,295	0,0
193	-7	3	4	1,38786	67,425	0,2
194	3	3	5	1,38689	67,478	0,0
195	10	0	2	1,38518	67,573	0,0
196	9	1	3	1,38371	67,654	0,0
197	0	2	7	1,38237	67,729	0,0
198	5	3	4	1,38055	67,831	0,2
199	-10	2	1	1,37556	68,110	0,0
200	-4	2	7	1,37504	68,139	0,0
201	-2	0	8	1,37356	68,223	0,5
202	-4	4	3	1,37318	68,245	0,0
203	-1	3	6	1,37305	68,252	0,0
